# *Clostridioides difficile* water-soluble zwitterionic lipoteichoic acid wsPS-III: structure, synthesis, and immunogenicity

**DOI:** 10.3389/fimmu.2026.1901286

**Published:** 2026-08-27

**Authors:** Zuchao Ma, Brittany Pequegnat, Douglas Hodgins, Martin Sagermann, Jeyabarathy Ganeshapillai, Sharif Arar, Jamie Dawson, Nolan W. Frame, Samantha Wheadon, Herbert Chow, Luis Arroyo, Mario A. Monteiro

**Affiliations:** 1 Department of Chemistry, University of Guelph, Guelph, ON, Canada; 2 Department of Pathobiology, University of Guelph, Guelph, ON, Canada; 3 Stellar Biotechnologies Inc., Port Hueneme, CA, United States; 4 Department of Clinical Studies, University of Guelph, Guelph, ON, Canada

**Keywords:** *Clostridioides difficile*, immunogenicity, polysaccharide, synthesis, zwitterionic

## Abstract

*Clostridioides difficile* polysaccharides (PSs) represent useful tools for the study of *C. difficile* pathogenesis. Water-soluble PS-I (pentaglycosyl-phosphate repeats) and PS-II (hexaglycosyl-phosphate repeats) and phenol-soluble lipoteichoic acid (LTA) PS-III have been previously identified. Most of the LTA PS-III repeats consist of two *N*-acetyl-glucopyranosamine (GlcNAc) units and glyceric acid (GroA), bridged by an exocyclic phosphodiester: →P-6)-α-D-GlcNAc-(1-3)-α-D-GlcNAc-[1-2-GroA]-(6→. Here, we describe a water-soluble PS from an equine *C. difficile* strain similar to PS-III, but with 6-substituted glucopyranosamine (GlcN) predominantly in the repeat: →P-6)-α-D-GlcN-(1-3)-α-D-GlcNAc-[1-2-GroA]-(6→ (coined wsPS-III). For analytical comparisons, the two saccharides, α-D-GlcN-(1-3)-α-D-GlcNAc-(1-2)-GroA and α-D-GlcNAc-(1-3)-α-D-GlcNAc-(1-2)-GroA, were chemically synthesized, and the transformation product, Gro-(1-P-6)-α-D-GlcNAc-(1-2)-GroA, was generated by Smith degradation. Pre-existing circulating IgG antibodies in horse sera due to natural exposure reacted with wsPS-III, and inoculation of rabbits with wsPS-III afforded IgG antibodies that reacted with *C. difficile*. It is postulated that the zwitterionic nature of wsPS-III is the driving force behind the observed solubility and immunogenicity. The glycans presented here will be useful in investigations concerning *C. difficile* biosynthetic pathways, virulence, antibiotics and vaccines.

## 1 Introduction

*Clostridioides difficile* (formerly *Clostridium difficile*) infections stand as one of the main challenges to healthcare systems (1). So far, the *C. difficile* exotoxins A and B have been the primary focus of research, including vaccine programs (2), but recent human trials showed that methodologies centered solely on such exotoxins are not enough to fully understand and prevent the full cycle of *C. difficile* pathogenesis (3). Therefore, studies and vaccines based on other *C. difficile* virulence factors, such as surface proteins and polysaccharides (PSs), are desirable.

*C. difficile* exposes specific immunogenic PSs, two water-soluble PSs akin to teichoic-acids, PS-I with a pentasaccharide-phosphate repeat made of rhamnose (Rha), glucose (Glc), and diester phosphate (P): →2)-α-D-Glc*p*-(1-P-4)-α-L-Rha*p*-(1–3)-β-D-Glc*p*-(1–4)-[α-L-Rha*p*-(1-3]-α-D-Glc*p*-(1→, and PS-II composed of hexasaccharide-phosphate repeats with Glc, mannose (Man), *N*-acetyl-galactosamine (GalNAc), and P: →3)-α-D-Man*p*-(1-P-6)-β-D-Glc*p*-(1–3)-β-D-Gal*p*NAc-(1–4)-α-D-Glc*p*-(1–4)-[β-D-Glc*p*-(1–3)]-β-D-Gal*p*NAc-(1→ (4, 5). Evidence of a third *C. difficile* PS (named PS-III) composed of *N*-acetyl-glucosamine (Glc*p*NAc), Glc, glycerol (Gro), and P was also obtained from water-insoluble pellet material (5). This structure was subsequently fully identified as a phenol-soluble lipoteichoic acid (LTA) with repeats made of P, 6- and 3,6-substituted Glc*p*NAc units, and 2-substituted glyceric acid (GroA), which were connected by a P through positions 6 of the GlcNAc units: →P-6)-α-D-GlcNAc-(1–3)-α-D-GlcNAc-[1-2-GroA]-6→ (6). Some repeats (~30%) contained 6-substituted glucosamine (GlcN) instead of 6-substituted GlcNAc: →P-6)-α-D-GlcN-(1–3)-α-D-GlcNAc-[1-2-GroA]-6→ (6, 7). Candela and coworkers have also described the possible presence of alanine in LTA PS-III (7).

*C. difficile* PS-I, PS-II, and PS-III quickly became the focus of conjugate vaccine investigations that used both native PSs and synthetically derived oligosaccharides (8–22). *C. difficile* PS-I, PS-II, and PS-III conjugates were shown to be immunogenic in animal models and effective in lowering colonization levels (23, 24). Reactions were seen between circulating human serum antibodies and PS-I, PS-II, and PS-III (10, 18). The main attention, however, turned to PS-II because, unlike PS-I, it has been found to be exposed by all *C. difficile* ribotypes (16). LTA PS-III is also a conserved *C. difficile* antigen, but removal of fatty acids is necessary to reach vaccine endotoxin limits. Anti-LTA PS-III antibodies were also non-specific as they cross-reacted with several commensal Clostridia species (20). Thus, most carbohydrate-based research efforts have employed the PS-II antigen, and indeed, a phase I human clinical trial (Idorsia Pharmaceuticals Ltd) using a synthetic PS-II vaccine (IDOR-1134-2831) has recently taken place.

Other research has determined that PS-II is essential for *C. difficile* survival (25, 26), a key player in the architecture of *C. difficile* biofilm and cell-wall protein scaffolds (27–30) and a target of antibiotic mechanisms (31, 32). Also, new biosynthetic pathways, such as that involved in the phosphorylation of hexasaccharide-phosphate PS-II, which uses a novel mannosyl-1-phosphotransferase in the synthesis of the phosphate bridge between Man and Glc […α-D-Man*p*-(1-P-6)-β-D-Glc*p* … ], have been identified (33).

*C. difficile* infections are also a burden in farm animals, especially high-value racehorses (34). During our efforts to develop a PS-II equine vaccine (35), extractions of glycans from an equine *C. difficile* strain consistently afforded a distinctive material (qualitative ^31^P NMR analysis) in the aqueous layer along with PS-II. ^1^H NMR analysis suggested that it could be related to the previously characterized phenol-soluble PS-III, but the new ^31^P resonance along with differences in the glycosyl anomeric (H-1) and GroA α-proton (H-2) regions suggested slight variations in structure, and thus, a closer look at this water-soluble material was warranted.

In this work, we identify a water-soluble PS akin to phenol-soluble PS-III, coined wsPS-III, in which the repeating blocks contain predominantly 6-substituted GlcN in place of 6-substituted GlcNAc: →P-6)-α-D-GlcN-(1–3)-α-D-GlcNAc-[1-2-GroA]-(6→. Shown here, too, are the chemical syntheses of the two related saccharide repeats, α-D-GlcN-(1–3)-α-D-GlcNAc-(1–2)-GroA and α-D-GlcNAc-(1–3)-α-D-GlcNAc-(1–2)-GroA, whose NMR data shed light on possible reasons behind the variability in GroA H-2 chemical shifts between the GlcN-(1-3) containing glycans and those with GlcNAc-(1-3). Lastly, a head-to-head assessment of PS-I, PS-II, and wsPS-III reactivity with naturally circulating IgG antibodies in healthy horse sera and the reaction of wsPS-III IgG antibodies raised in rabbits with *C. difficile* are described.

## 2 Materials and methods

### 2.1 Growth of *C. difficile* and isolation of wsPS-III

An equine *C. difficile* isolate (A^+^B^+^CDT^+^) was cultivated for 5 days in dialysis tubing (molecular weight cutoff, 100 kDa) suspended in brain heart infusion broth. The contents of the dialysis bags were centrifuged at 10,000 rpm for 30 min, and the cell pellets were pooled for polysaccharide extraction.

*C. difficile* cell paste was added to a 100-mL solution containing 60 mL of water and 40 mL of phenol and then stirred for 3 h at 70 °C. The solution was cooled in an ice bath for 16 h that resulted in the separation of the aqueous and phenol layers. The phenol and aqueous solutions were dialyzed for 2 days against water, lyophilized, and treated with RNAse. The PS-II and wsPS-III preparations were obtained by fractionation of crude material through a Bio-Gel P-2 column.

### 2.2 Smith degradation

Native wsPS-III (2 mg) was dissolved in a 5-mL solution of 0.1 M sodium acetate with 0.04 M of NaIO_4_ at pH 4 and left at 4°C in the dark for 3 days. The oxidized material was dialyzed against running water (cutoff 2,000 Da) and lyophilized. The oxidized material was then reduced with NaBD_4_ at room temperature for 2 days, followed by treatment with methanolic acetic acid, dialyzed against running water, and lyophilized. The reduced material was treated with 1 M of TFA at 45 °C for 1 h and in one case purified by dialysis (cutoff 1,000 Da) in sitting water and in the other by size exclusion chromatography (G-25) with water as eluent.

### 2.3 Enzyme-linked immunosorbent assay and immunofluorescence microscopy

Serum IgG antibodies to PS-I, PS-II, and wsPS-III were assayed using enzyme-linked immunosorbent assays (ELISA). PSI, PS-II, and wsPS-III, 0.02 mg/mL in PBS pH 7.4, were coated to polystyrene 96-well plates (Thermo Scientific, Rochester, NY, catalog number 269620) at 100 µL per well at 37 °C for 2 h. Plates were washed with PBS wash solution containing 0.5% fish skin gelatin (Sigma, St. Louis, MO) and 0.05% Tween 20 (Sigma). Individual horse sera diluted 1/50 in sample diluent consisting of PBS containing 0.3 M of sodium chloride, 0.5% fish skin gelatin, and 1.5% Tween 20 were added to the wells (100 µL per well) and incubated at 37 °C for 1 h. Plates were washed with PBS wash solution; bound IgG was detected using goat anti-horse IgG (H and L chain specific) horseradish peroxidase conjugate [Kirkegaard and Perry Laboratories (KPL), Gaithersburg, MD]. ABTS (2,2′-azino-di(3-ethylbenzthiazoline-6-sulfonate), KPL) was used for color development. Plates were read on a BioTek PowerWave XS2 (Winooski, VT) plate reader at 405 nm after 50 min of incubation. Antibody concentrations were expressed as the ratio (S/P ratio) of the optical density for the serum sample to the optical density for a positive control serum also diluted 1/50. Sera collected from foals before colostral intake were used as negative controls. New Zealand rabbits were immunized four times, every 10 days, subcutaneously with 200 μg of wsPS-III and complete Freund’s adjuvant. After 35 days, serum was extracted and used in the immunofluorescence microscopy study. The *C. difficile* strain used in the immunofluorescence microscopy study belonged to ribotype 027 and the anti-rabbit IgG secondary antibody was ABCAM FITC.

### 2.4 Mass spectrometry and nuclear magnetic resonance spectroscopy

Matrix-assisted laser desorption/ionization time-of-flight mass spectrometry (MALDI-ToF-MS) analysis was performed using a Bruker autoflex maX instrument (Bruker Daltonik, Bremen, Germany) equipped with a Smartbeam II solid-state laser. The instrument was operated in linear and reflective positive ion modes, within detection ranges of 0–1,000 *m*/*z*. Data were acquired through the accumulation of laser shots, and an ion-source voltage of 19 kV was applied. Methylation of material was carried out using the same protocol described for permethylated alditol acetates. A Super 2,5-dihydroxybenzoic acid (DHB) matrix (Sigma Aldrich, 50862) was prepared to a concentration of 20 mg/mL in 50% (v/v) acetonitrile in H_2_O. Methylated wsPS-III was resuspended in the Super-DHB matrix, where 2 μL of the resuspension was spotted and air-dried to its respective well on a Bruker MALDI MTP 384 target plate (polished steel BC microplate, Part No. 8280781). The data were acquired using Bruker Compass flexControl software and viewed/analyzed on Bruker Compass flexAnalysis software, both versions 3.4.

The 1D ^1^H NMR experiments were conducted on a Bruker Avance III 400 MHz NMR spectrometer with an enhanced-sensitivity Prodigy probe at 298K. 1D selective total correlation spectroscopy (TOCSY), 1D selective nuclear Overhauser effect spectroscopy (NOESY), 2D heteronuclear single quantum coherence spectroscopy (HSQC), and 2D homonuclear multiple bond correlation spectroscopy (HMBC) were conducted on the Bruker Avance III UltraShieldTM 600 MHz NMR spectrometer equipped with a 5-mm TCI Cryoprobe under varying conditions (295K and 315K). All data analysis was completed using the software program Bruker TopSpin 4.1.4. All NMR samples were prepared with five deuterium exchanges through multiple lyophilizations in deuterium oxide (D_2_O). NMR samples were referenced to acetone (*δ*_H_ 2.22 ppm and *δ*_C_ 31.7).

### 2.5 Chemical synthesis

#### 2.5.1 General methods

All chemicals were purchased from commercial suppliers and used as received except for AgOTf, which was weighed immediately before use under rigorously anhydrous conditions, and anhydrous CH_2_Cl_2_, to which activated 4-Å molecular sieves (activated by heating and dried under reduced pressure) were added and stored for 48 h prior to use. Thin layer chromatography (TLC) was carried out on TLC silica gel F_254_. Sugar compounds were visualized by UV light or by charring with 5% H_2_SO_4_ in ethanol. Flash chromatography was performed with silica gel P60 (43–60 μm, 230–400 mesh). ^1^H NMR and ^13^C NMR spectra were recorded on Bruker 400 and 100 MHz spectrometers, respectively. The proton signal of residual, non-deuterated solvent (*δ* 7.24 ppm for CHCl_3_) was used as an internal reference for ^1^H NMR spectra. For ^13^C spectra, the chemical shifts are reported relative to the solvent (*δ* 77.1 ppm for CDCl_3_). Chemical shifts are reported in parts per million (ppm). Coupling constants are reported in Hertz (Hz). The following abbreviations are used to indicate the multiplicities: s, singlet; d, doublet; t, triplet; m, multiplet. Optical rotations were measured on a Rudolph Research Autopol IV automatic polarimeter and concentration (*c*) is expressed in g/100 mL. High-resolution mass spectra were recorded at the Mass Spectrometry Facility, University of Guelph.

#### 2.5.2 3,4,6-Tri-O-acetyl-2-p-methoxybenzylideneamino-2-deoxy-α β-D-glucopyranosyl trichloroacetimidate

Glucosamine hydrochloride (5.0 g, 23.26 mmol) and *p*-anisaldehyde (2.85 mL, 23.5 mmol) were stirred in 1.0 M of sodium hydroxide (24 mL) for 1 h. The reaction mixture was cooled in an ice bath for 1 h, and the resulting precipitate was collected by filtration and dried under high vacuum overnight to afford crude product 2. Acetic anhydride (15 mL, 158.8 mmol, 6.8 eq.) and pyridine (20 mL) were added to product 2, and the reaction mixture was stirred at room temperature for 16 h. The mixture was then concentrated under reduced pressure to afford product 3, to which saturated ammonia in methanol/toluene (1:3, v/v) was added. The reaction was monitored by TLC and was completed after 2.5 h at room temperature. The solvents were removed under reduced pressure to give a residue, to which dichloromethane (50 mL), trichloroacetonitrile (5.0 mL, 49.0 mmol, 2.1 eq.), and potassium carbonate (3.5 g, 25.36 mmol, 1.1 eq.) were added. The reaction mixture was stirred at room temperature for 16 h and filtered through a pad of Celite. The filtrate was concentrated and purified by flash column chromatography on silica gel (hexane/ethyl acetate, 3:1, containing 1% triethylamine) to furnish donor 4 as an oily solid (8.2 g, α-anomer:β-anomer = 29:71 by NMR, 62% over four steps). The ¹H and ¹³C NMR and MS data of the α-anomer were consistent with the reported data (36). For the β-anomer: ^1^H NMR (400 MHz, CDCl_3_): *δ* 8.61 (s, 1H, OCN*H*CCl_3_), 8.21 (s, 1H, NC*H*PhOCH_3_), 7.61 (d, *J* = 8.5 Hz, 2H, Ph), 6.86 (d, *J* = 8.5 Hz, 2H, Ph), 6.01 (d, *J* = 9.0 Hz, 1H, H-1), 5.51 (t, *J* = 9.5 Hz, 1H, H-3), 5.20 (t, *J* = 9.5 Hz, 1H, H-4), 4.40–4.36 (m, 1H, H-6a), 4.18–4.14 (m, 1H, H-6b), 4.02–3.97 (m, 1H, H-5), 3.81 (s, 3H, OCH_3_), 3.61 (t, *J* = 9.0 Hz, 1H, H-2), 2.09 (s, 3H, COCH_3_), 2.04 (s, 3H, COCH_3_), 1.89 (s, 3H, COCH_3_). ^13^C NMR (100 MHz, CDCl_3_): *δ* 170.8, 169.9, 169.8, 165.2, 162.2, 160.7, 130.2, 128.4, 114.0, 96.9, 73.4, 73.3.72.8, 68.1, 61.9, 55.4, 20.8, 20.7, 20.6. HRMS (MALDI): Calcd. for C_22_H_25_Cl_3_N_2_O_9_ [M+H]^+^: 567.0698, found: 567.0683.

#### 2.5.3 Methyl-(2R)-2-hydroxy-trityloxypropanoate

To a solution of D-glyceric acid calcium salt dihydrate (5) (0.50 g, 1.75 mmol) in methanol (30 mL), acetyl chloride (1.5 mL) was added slowly at 0°C, and the solution was stirred at room temperature for 1 h. The reaction mixture was concentrated under reduced pressure to afford crude glyceric acid methyl ester 6 as a foamy residue, which was dried under high vacuum overnight and used in the next step without further purification. Pyridine (10 mL) and trityl chloride (1.0 g, 3.59 mmol, 2.1 eq.) were added, and the reaction mixture was stirred at 55°C for 24 h. The solvent was removed under reduced pressure, and the resulting residue was purified by flash column chromatography (ethyl acetate) to afford product 7 as a colorless oil (0.42 g, 66% over two steps). ^1^H NMR (400 MHz, CDCl_3_): *δ* 7.42~7.19 (m, 15H, 3 C_6_H_5_), 4.28~4.24 (m, 1H, H-2), 3.74 (s, 3H, OCH_3_), 3.46 (dd, *J* = 12.5, 3.0 Hz, 1H, H-3a), 3.34 (dd, *J* = 12.5, 3.5 Hz, 1H, H-3b), 3.15 (d, *J* = 12 Hz, 1H, OH). ^13^C NMR (100 MHz, CDCl_3_): *δ* 173.6, 143.6, 128.6, 128.0, 127.9, 127.3, 127.2, 86.4, 70.8, 65.3, 52.5. HRMS (MALDI): Calcd. for C_23_H_22_O_4_ [M+H]^+^: 363.1591, found: 363.1575.

#### 2.5.4 3,4,6-Tri-O-acetyl-2-amino-2-deoxy-α-D-glucopyranosyl-(1→2)-methyl (2R)-3-trityloxypropanoate

Glyceric acid acceptor **7** (180 mg, 0.50 mmol), glucosamine donor **4** (300 mg, 0.53 mmol, 1.1 eq.), silver triflate (13.7 mg, 0.05 mmol, 0.1 eq.), and activated molecular sieves were dried under high vacuum for 2 h, then dichloromethane (6 mL) was added, and the reaction mixture was stirred at room temperature for 48 h. The reaction was quenched with triethylamine (10 μL), filtered, and concentrated under reduced pressure to afford a residue, which was treated with 80% aqueous acetic acid (2 mL) at 80 °C for 2.5 h. The mixture was concentrated under reduced pressure, and the resulting residue was purified by silica gel column chromatography (hexane/ethyl acetate, 1:5) to afford product 8 as a white solid (217 mg, 67%). ^1^H NMR (400 MHz, CDCl_3_): *δ* 7.51–7.18 (m, 17H, 3 C_6_H_5_, NH_2_), 5.04 (t, *J* = 9.5 Hz, 1H, H-4_GlcN_), 4.81 (d, *J* = 2.8 Hz, 1H, H-1_GlcN_), 4.36–4.32 (m, 1H, H-2_GroA_), 4.16–3.92 (m, 5H, H-2_GlcN_, H-3_GlcN_, H-5_GlcN_, 2 H-6_GlcN_), 3.72 (s, 3H, OCH_3_), 3.50–3.4 (m, 2H, 2 H-3_GroA_), 2.12 (s, 3H, COCH_3_), 2.08 (s, 3H, COCH_3_), 2.01 (s, 3H, COCH_3_). ^13^C NMR (100 MHz, CDCl_3_): *δ* 173.4, 171.6, 170.8, 170.0, 143.3, 128.5, 128.0, 127.4, 97.7, 86.9, 75.9, 72.9, 71.0, 68.5, 64.4, 61.8, 55.1, 52.8, 22.9, 21.0, 20.8. HRMS (MALDI): Calcd. for C_35_H_39_NO_11_ [M+H]^+^: 650.2596, found: 650.2580.

#### 2.5.5 3,4,6-Tri-O-acetyl-2-acetamido-2-deoxy-α-D-glucopyranosyl-(1→2)-methyl (2R)-3-trityloxypropanoate

To a solution of compound 8 (158 mg, 0.24 mmol) in pyridine (0.5 mL) was added acetic anhydride (0.5 mL, 5.3 mmol, 22 eq.). The reaction mixture was stirred at room temperature for 5 h and then concentrated under reduced pressure. The residue was passed through a short silica gel column (hexane/ethyl acetate, 1:4) to afford product 9 as a white solid (165 mg, 0.23 mmol, 98%). ^1^H NMR (400 MHz, CDCl_3_): *δ* 7.44~7.20 (m, 15H, 3 C_6_H_5_), 6.34 (d, *J* = 9.0 Hz, 1H, NH), 5.33 (t, *J* = 9.5 Hz, 1H, H-3_GlcN_), 5.15 (t, *J* = 9.5 Hz, 1H, H-4_GlcN_), 4.81 (d, *J* = 2.9 Hz, 1H, H-1_GlcN_), 4.41–4.32 (m, 2H, H-2_GroA_, H-2_GlcN_), 4.18–4.14 (m, 1H, H-5_GlcN_), 4.09–3.95 (m, 2H, 2 H-6_GlcN_), 3.68 (s, 3H, OCH_3_), 3.50–3.42 (m, 2H, 2 H-3_GroA_), 2.05 (s, 3H, COCH_3_), 2.03 (s, 3H, COCH_3_), 2.00 (s, 3H, COCH_3_), 1.95 (s, 3H, COCH_3_). ^13^C NMR (100 MHz, CDCl_3_): *δ* 171.1, 170.8, 170.7, 170.3, 169.3, 143.3, 128.8, 128.6, 128.0, 127.8 127.4, 97.4, 87.2, 71.3, 68.4, 67.8, 64.5, 61.6, 52.5, 51.4, 45.2, 23.2, 20.9, 20.8, 20.6. HRMS (MALDI): Calcd. for C_37_H_41_NO_12_ [M+H]^+^: 692.2702, found: 692.2686.

#### 2.5.6 2-Acetamido-2-deoxy-α-D-glucopyranosyl-(1→2)-methyl (2R)-3-trityloxypropanoate

To a solution of compound 9 (140 mg, 0.20 mmol) in methanol (2 mL) was added potassium carbonate (25 mg), and the reaction mixture was stirred for 3 h at room temperature. The mixture was then concentrated under reduced pressure, and the residue was purified by silica gel column chromatography (ethyl acetate/methanol, 5:1) to afford product 10 as a white solid (88.1 mg, 77%). ^1^H NMR (400 MHz, CDCl_3_): *δ* 7.45–7.18 (m, 16H, 3 C_6_H_5_, NH), 4.78 (d, *J* = 2.8 Hz, 1H, H-1_GlcN_), 4.35–4.31 (m, 1H, H-2_GroA_), 4.02–3.94 (m, 1H, H-2_GlcN_), 3.84 (t, *J* = 9.2 Hz, 1H, H-3_GlcN_), 3.80–3.55 (m, 7H, H-4_GlcN_, H-5_GlcN_, 2 H-6_GlcN_, OCH_3_), 3.51–3.35 (m, 2H, 2 H-3_GroA_), 2.11 (s, 3H, COCH_3_). ^13^C NMR (100 MHz, CDCl_3_): *δ* 173.4, 171.8, 143.4, 128.5, 128.0, 127.3, 98.0, 86.7, 75.8, 74,5, 72.1, 71.2, 64.5, 61.7, 54.5, 52.6, 23.0. HRMS (MALDI): Calcd. for C_31_H_35_NO_9_ [M+H]^+^: 566.2385, found: 566.2368.

#### 2.5.7 2-Acetamido-2-deoxy-6-O-trityl-α-D-glucopyranosyl-(1→2)-methyl (2R)-3-trityloxypropanoate

Compound 10 (85.0 mg, 0.15 mmol) and trityl chloride (180 mg, 0.79 mmol, 5.3 eq.) were dissolved in pyridine (2 mL), and the reaction mixture was stirred at 55 °C for 48 h. The solvent was removed under reduced pressure, and the resulting residue was purified by silica gel column chromatography (hexane/ethyl acetate, 1:1) to afford product 11 as a white solid (78.9 mg, 65%). ^1^H NMR (400 MHz, CDCl_3_): *δ* 7.56–7.19 (m, 31H, 6 C_6_H_5_, NH), 4.82 (d, *J* = 2.5 Hz, 1H, H-1_GlcN_), 4.39–4.35 (m, 1H, H-2_GroA_), 4.07–4.01 (m, 1H, H-2_GlcN_), 3.91–3.81 (m, 2H, H-3_GlcN_, H-5_GlcN_), 3.75–3.69 (m, 4H, H-4_GlcN_, OCH_3_), 3.53–3.19 (m, 4H, 2 H-3_GroA_, 2 H-6_GlcN_), 2.15 (s, 3H, COCH_3_). ^13^C NMR (100 MHz, CDCl_3_): *δ* 173.5, 171.9, 143.4, 143.4, 128.7, 128.5, 128.0, 127.8, 127.2, 127.0, 97.7, 86.7, 86.5, 75.4, 75.2, 72.0, 71.4, 64.6, 62.8, 54.9, 52.6, 23.0. HRMS (MALDI): Calcd. for C_50_H_49_NO_9_ [M+H]^+^: 808.3480, found: 808.3443.

#### 2.5.8 2-p-Methoxybenzylideneamino-2-deoxy-α-D-glucopyranosyl-(1→3)-2-acetamido-2-deoxy-6-O-trityl-α-D-glucopyranosyl-(1→2)-methyl (2R)-3-trityloxypropanoate

Acceptor 11 (50.0 mg, 0.062 mmol), glucosamine donor 4 (70.0 mg, 0.12 mmol, 2.1 eq.), silver triflate (2.0 mg, 7.8 μmol, 0.13 eq.), and activated molecular sieves were dried under high vacuum for 2 h. Dichloromethane (2 mL) was added, and the reaction mixture was stirred at room temperature for 16 h. The reaction was quenched with triethylamine (2 μL), filtered, and concentrated under reduced pressure. The resulting residue was purified by silica gel column chromatography (hexane/ethyl acetate, 1:1) to afford product 12 as a white solid (48.9 mg, 65%). ^1^H NMR (400 MHz, CDCl_3_): *δ* 8.15 (s, 1H, NC*H*PhOCH_3_), 7.62~6.73 (m, 34H, 6 C_6_H_5_, C_6_H_4_), 6.64 (d, *J* = 10.0 Hz, 1H, NH), 6.35 (s, 1H, OH), 5.51 (t, *J* = 10.5 Hz, 1H, H-3_GlcN’_), 5.14 (t, *J* = 10.5 Hz, 1H, H-4_GlcN’_), 5.04 (d, *J* = 2.8 Hz, 1H, H-1_GlcN’_), 4.82 (d, *J* = 2.9 Hz, 1H, H-1_GlcN_), 4.41~4.18 (m, 5H, H-2_GroA_, H-2’, H-5_GlcN’_, 2 H-6_GlcN’_), 3.95~3.85 (m, 2H, H-4_GlcN_, H-5_GlcN_), 3.80 (s, 3H, OCH_3_), 3.72~3.62 (m, 4H, H-3_GlcN_, OCH_3_), 3.55~3.46 (m, 2H, H-2_GlcN’_, H-3a_GroA_), 3.38~3.32 (m, 2H, H-3b_GroA_, H-6a_GlcN_), 3.21 (dd, *J* = 10.5, 6.5 Hz, 1H, H-6b_GlcN_), 2.14 (s, 3H, COCH_3_), 2.08 (s, 3H, COCH_3_), 2.01 (s, 3H, COCH_3_), 1.82 (s, 3H, COCH_3_). ^13^C NMR (100 MHz, CDCl_3_): *δ* 171.1, 170.8, 1170.7, 170.3, 169.5, 166.3, 162.7, 144.2, 143.4, 131.0, 128.8, 128.4, 127.9, 127.7, 127.2, 127.1, 126.8, 114.4, 103.0, 98.6, 87.9, 86.5, 86.3, 75.1, 73.3, 72.7, 71.1, 70.2, 69.0, 68.2, 64.8, 63.0, 61.6, 55.5, 52.4, 51.9, 45.5, 23.7, 20.9, 20.8, 20.6. HRMS (MALDI): Calcd. for C_70_H_72_N_2_O_17_ [M+Na]^+^: 1,235.4723, found: 1,235.4705.

#### 2.5.9 2-Amino-2-deoxy-α-D-glucopyranosyl-(1→3)-2-acetamido-2-deoxy-α-D-glucopyranosyl-(1→2)-(2R)-3-hydroxypropanoic acid

Protected disaccharide 12 (18.0 mg, 14.8 μmol) was dissolved in 80% aqueous acetic acid (0.5 mL) and heated at 80 °C for 3 h. The reaction mixture was concentrated to dryness under reduced pressure. Sodium methoxide in methanol (0.5 M, 0.5 mL) was added, and the solution was stirred at room temperature for 4 h, then neutralized with acetic acid (0.1 mL). The mixture was concentrated under reduced pressure and purified by P2 gel column chromatography (water) to afford target compound 13 as a white solid (4.2 mg, 60%). [α]_D_^20^ = +11.1° (c = 0.02, H_2_O). ^1^H NMR (400 MHz, D_2_O): *δ* 5.62 (d, 1H, *J* = 3.9 Hz, H-1_GlcN_), 4.85 (d, 1H, *J* = 3.3 Hz, H-1_GlcNAc_), 4.14 (dd, 1H, *J* = 7.0, 3.5 Hz, H-2_GroA_), 4.09–4.04 (m, 2H, H-2_GlcNAc_, H-3_GlcNAc_), 3.89–3.69 (m, 14H, H-3_GlcN_, 2 H-6_GlcNAc_, H-4_GlcNAc_, H-5_GlcNAc_, 2 H-6_GlcN_, 2 H-3_GroA_), 3.59–3.56 (m, 1H, H-5_GlcN_), 3.50 (t, 1H, *J* = 10.0 Hz, H-4_GlcN_), 3.27 (dd, 1H, *J* = 10.0, 3.9 Hz, H-2_GlcN_), 2.03 (s, 3H, COCH_3_). ^13^C NMR (100 MHz, D_2_O): *δ* 176.9, 174.4, 96.6, 96.0, 79.1, 76.7, 72.5, 72.1, 70.7, 69.2, 68.7, 62.9, 59.9, 59.6, 54.0, 51.9, 22.1. HRMS (MALDI): Calcd. for C_17_H_30_N_2_O_13_ [M+H]^+^: 471.1821, found: 471.1808.

#### 2.5.10 2-Acetamido-2-deoxy-α-D-glucopyranosyl-(1→3)-2-acetamido-2-deoxy-α-D-glucopyranosyl-(1→2)-(2R)-3-hydroxypropanoic acid

Protected disaccharide **12** (28.1 mg, 23.2 μmol) was dissolved in 80% aqueous acetic acid (0.5 mL) and heated at 80 °C for 3 h. The solvents were removed under reduced pressure, and acetic anhydride (0.2 mL) and pyridine (0.2 mL) were added. The reaction mixture was stirred for 5 h and then concentrated to dryness. Sodium methoxide in methanol (0.5 M, 0.5 mL) was added, and the mixture was stirred at room temperature for 4 h, then neutralized with acetic acid (0.1 mL). The reaction mixture was concentrated under reduced pressure and purified by P2 gel column chromatography (water) to afford target compound 14 as a white solid (6.5 mg, 55%). [α]_D_^20^ = +5.9° (c = 0.02, H_2_O). ^1^H NMR (400 MHz, D_2_O): *δ* 5.34 (d, 1H, *J* = 3.9 Hz, H-1_GlcNAc_), 4.90 (d, 1H, *J* = 3.9 Hz, H-1_GlcNAc’_), 4.27 (t, 1H, *J* = 4.0 Hz, H-2_GroA_), 4.06 (dd, 1H, *J* = 10.0, 3.9 Hz, H-2_GlcNAc_), 3.92 (t, 1H, *J* = 10.0 Hz, H-3_GlcNAc_), 3.88–3.55 (m, 11H, H-2_GlcNAc’_, H-3_GlcNAc’_, H-4_GlcNAc’_, H-5_GlcNAc’_, 2 H-6_GlcN’_, H-5_GlcNAc_, 2 H-6_GlcNAc_, 2 H-3_GroA_), 3.50 (t, 1H, *J* = 10.0 Hz, H-4_GlcNAc’_), 2.01 (s, 3H, COCH_3_), 2.02 (s, 3H, COCH_3_). ^13^C NMR (100 MHz, D_2_O): *δ* 174.7, 174.3, 174.2, 97.7, 96.7, 77.5, 76.2, 72.3, 72.2, 70.6, 70.5, 69.2, 62.2, 60.0, 59.9, 53.6, 51.9, 21.9, 21.8. HRMS (MALDI): Calcd. for C_17_H_30_N_2_O_13_ [M+H]^+^: 513.1926, found: 513.2008.

## 3 Results

### 3.1 Structure of *C. difficile* water-soluble wsPS-III

**Figure 1:**
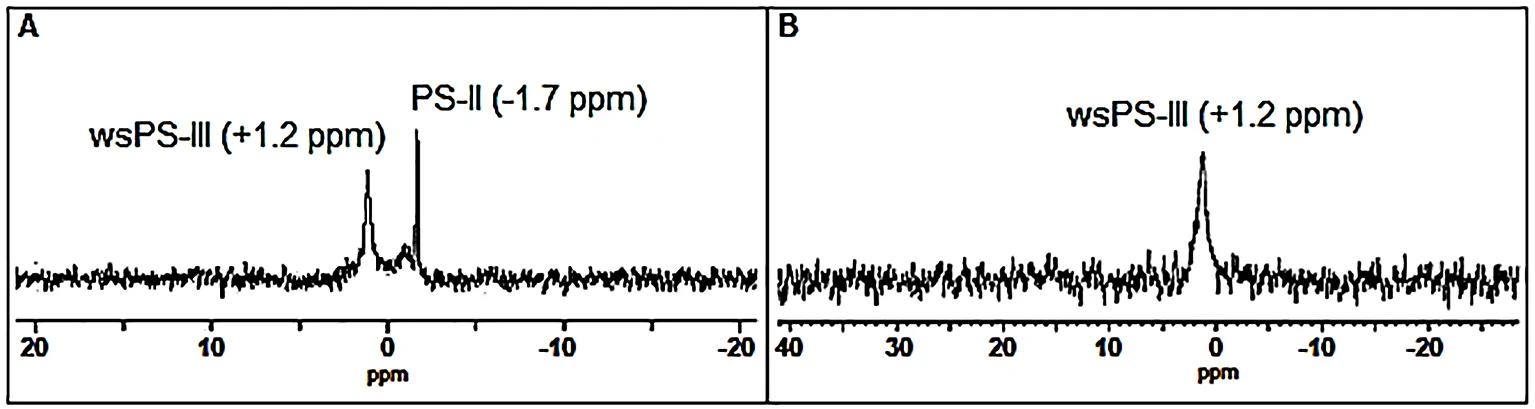
The ^31^P NMR spectrum **(A)** of the crude aqueous carbohydrate preparation showing resonances at −1.7 ppm of PS-II and that of wsPS-III at +1.2 ppm; and the ^31^P NMR spectrum **(B)** of isolated wsPS-III showing a sole resonance at +1.2 ppm.

**Figure 2:**
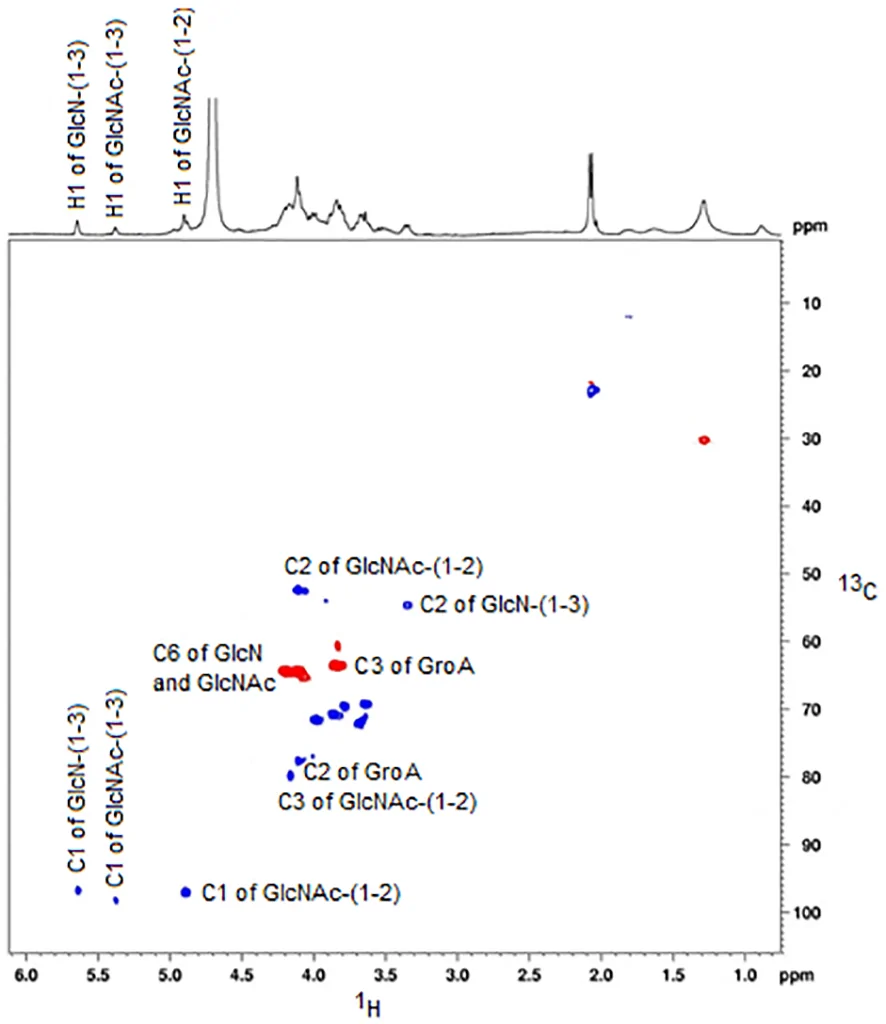
The 1D ^1^H NMR and 2D ^1^H-^13^C HSQC NMR spectra of native wsPS-III.

A key indicator that may be used for the quick detection of *C. difficile* water-soluble PS-I and PS-II and phenol-soluble PS-III is their specific phosphorus resonances at δP −0.9 (4), δP −1.7 (4), and δP −0.5 (6), respectively. In this study, the aqueous phase (from hot water-phenol treatment of *C. difficile* cells) afforded material whose ^31^P NMR showed two resonances (**Figure 1A**), one at δP −1.7 for PS-II and another at δP +1.2. That the entity responsible for the resonance at δP +1.2 was independent of PS-II was shown by its isolation after size exclusion chromatography (**Figure 1B**). An initial ^1^H NMR analysis (**Table 1**; **Figure 2**) revealed that this material was comparable to the previously identified phenol-soluble PS-III, with the two anomeric (H-1) resonances at δH 5.61 and 5.36 matching those of α-D-GlcN-(1-3) at δH 5.62 and α-D-GlcNAc-(1-3) at δH 5.33 of PS-III and a third anomeric at δH 4.90 slightly upfield of that associated with GlcNAc-(1-2) at δH 4.98 of PS-III.

**Table 1:** ^1^H NMR chemical shifts of H-1 of GlcN-(1-3), GlcNAc-(1-3), and GlcNAc-(1-2) and H-2 of GroA.

Glycan	H-1 GlcN-(1-3)	H-1 GlcNAc-(1-3)	H-1 GlcNAc-(1-2)	H-2 GroA
wsPS-III major repeat (this work) →P-6)-**α-GlcN-(1-3)**-α-GlcNAc-[1-2-GroA]-(6→	5.61	*Minor* 5.36	4.90	4.11
PS-III major repeat (6, 7) →P-6)-**α-GlcNAc-(1-3)**-α-GlcNAc-[1-2-GroA]-(6→	*Minor* 5.62	5.33	4.98	4.41
Smith degradation products (this work) Gro-(1-P-6)-α-GlcNAc-(1–2)-GroA (dialyzed) Gro-(1-P-6)-α-GlcNAc-(1–2)-GroA (desalted)			4.95 5.00	4.27 4.42
Synthetic with GlcN-(1–3) (this work) **α-GlcN-**(1–3)-α-GlcNAc-(1–2)-GroA	5.62		4.85	4.14
Synthetic with GlcNAc-(1–3) (this work) **α-GlcNAc-**(1–3)-α-GlcNAc-(1-2)-GroA		5.34	4.90	4.27
Synthetic with GlcNAc-(1-3) (37) **α-GlcNAc-**(1–3)-α-GlcNAc-(1–2)-GroA		5.39	4.90	4.26
Synthetic with GlcNAc-(1–3) (15) **α-GlcNAc-**(1–3)-α-GlcNAc-(1–2)-GroA		5.37	5.01	4.46

Due to the ^1^H NMR spectral similarities with the phenol-soluble PS-III, this water-soluble material was named wsPS-III. However, a striking difference between the NMR spectra of wsPS-III and PS-III was the relative intensities of the three anomeric resonances. The two dominant anomeric resonances in wsPS-III (**Figure 2**) were those belonging α-GlcN-(1-3) at δH 5.61 and α-GlcNAc-(1-2) at δH 4.90, whereas in PS-III (Reid et al., 2012), those of α-GlcNAc-(1-3) at δH 5.33 and α-GlcNAc-(1-2) at δH 4.98 were the major anomeric proton resonances. This pointed to the fact that the major disaccharide component in wsPS-III was α-GlcN-(1-3)-α-GlcNAc-(1-2), and not α-GlcNAc-(1-3)-α-GlcNAc-(1-2) as found in the phenol-soluble PS-III (6, 7).

The anomeric carbon (C-1) NMR resonances of wsPS-III resonated at δC 97.1 for α-GlcN-(1-3), δC 98.5 for α-GlcNAc-(1-3), and δC 97.2 for α-GlcNAc-(1-2) (**Figure 2**). Taking advantage of the fact that H-2 of α-GlcN resonated alone at δH 3.38, protons H-3 to H-6 belonging to α-GlcN were fully assigned, with H-3 at δH 3.79, H-4 at δH 3.63, H-5 at δH 3.69, and H-6,6′ at δH 4.10, 4.20 (**Appendix A**). Of the other units, only H-2 (δH 4.14), H-3 (δH 4.16), and H-4 (δH 3.98) of α-GlcNAc-(1–2) and H-2 (δH 3.91) of the minor α-GlcNAc-(1-3) could be confidently assigned. With knowledge about the ring proton resonances, the ring carbon resonances for GlcN-(1-3) were assigned: C-2 at δC 55.1, C-3 at δC 69.9, C-4 at δC 69.4, and C-5 at δC 72.1 and methylene C-6 at δC 64.5 (**Figure 2**); and the resonances for C-2, C-3, and C-4 of GlcNAc-(1-2) at δC 52.6, δC 80.0, and δC 71.8, respectively, and C-2 of GlcNAc-(1-3) at δC 54.0 (**Figure 2**). The methylene resonances associated with the fatty acids at the reducing-end terminus of wsPS-III could be seen in the upfield region at δH 1.30 and δC 30.5.

**Figure 3:**
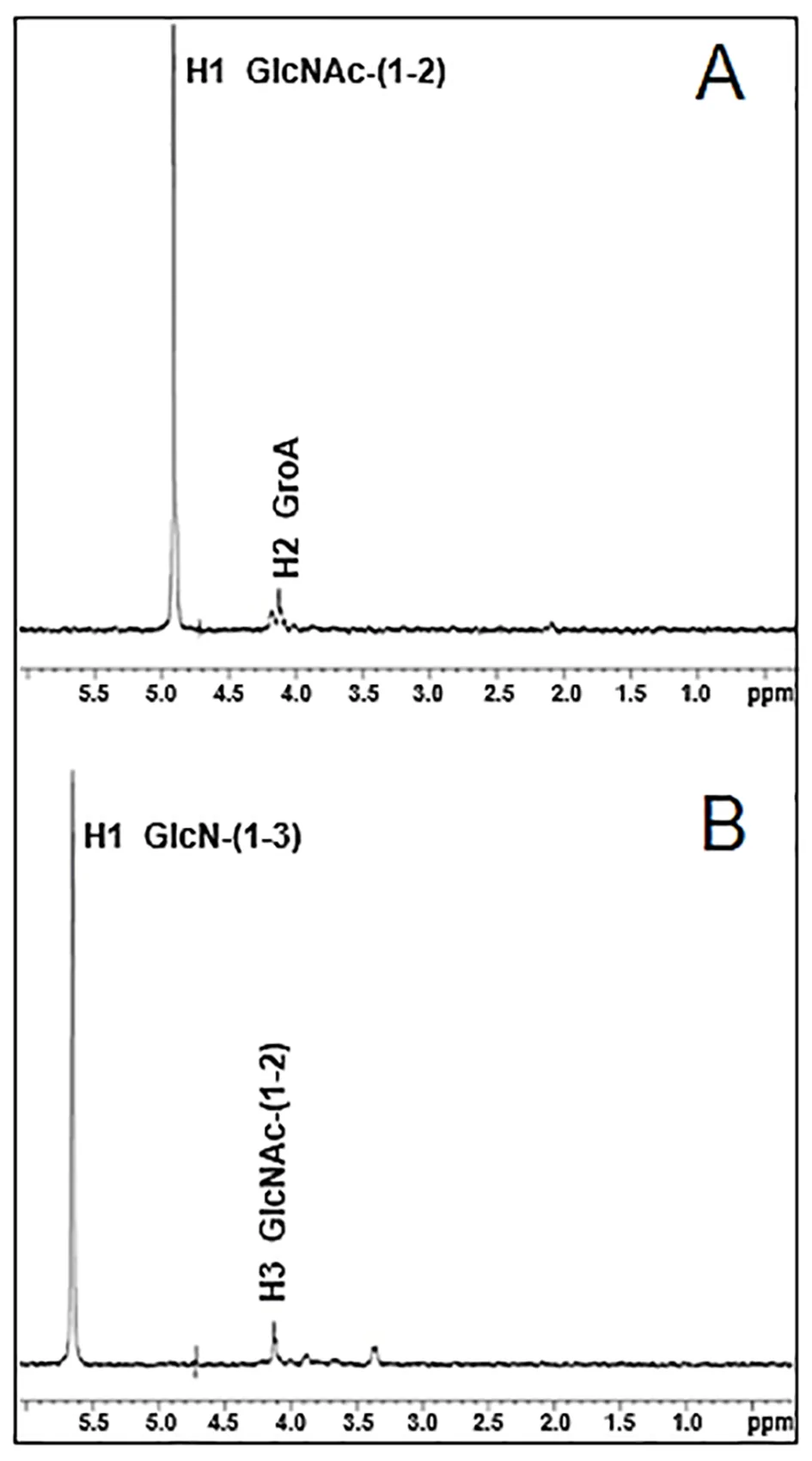
1D ^1^H-^1^H NOESY NMR spectra of wsPS-III showing inter-space connectivities between **(A)** H-1 of GlcNAc-(1-2) and H-2 of GroA for a GlcNAc-(1-2)-GroA linkage and between **(B)** H-1 of GlcN-(1-3) and H-3 of GlcNAc-(1-2) for a GlcN-(1-3)-GlcNAc-(1-2) linkage.

Another difference between wsPS-III and PS-III ^1^H NMR spectra was the chemical shift of the H-2 resonance of GroA, in PS-III resonated at δH 4.41 (6), but in wsPS-III, it was detected more upfield at δH 4.11 (**Figure 2**). That the resonance at δH 4.11 belonged to H-2 of the 2-substituted GroA in a GlcNAc-(1–2)-GroA linkage was shown by an inter-nOe connectivity to H-1 of α-GlcNAc-(1–2) (**Figure 3A**). The (1–3) linkage in GlcN-(1–3)-GlcNAc-(1–2) was also identified by an inter-nOe connectivity (**Figure 3B**) between H-1 of GlcN-(1–3) and H-3 (δH 4.15) of GlcNAc-(1-2). The expected intra-nOe connectivities between H-1 and H-2 of both α-GlcNAc-(1-2) and α-GlcN-(1-3) were also observed at δH 4.14 and δH 3.38, respectively (**Figures 3A, B**).

**Figure 4:**
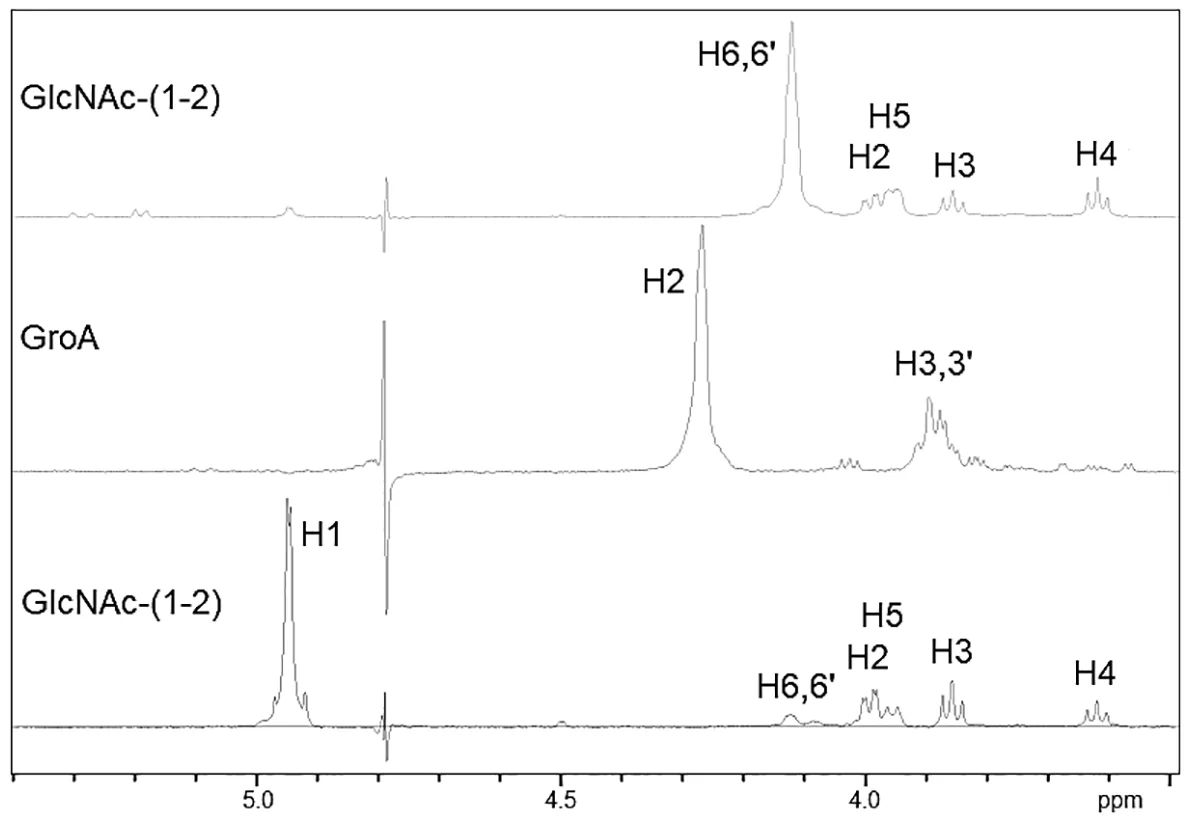
^1^H-^1^H 1D TOCSY NMR spectra of the Smith degradation product, ^2^H-Gro-(1-P-6)-α-GlcNAc-(1-2)-GroA, showing the proton resonances of GlcNAc-(1-2), irradiation of H6,6′ (top spectra) and H-1 (bottom spectra), and proton resonances of GroA (middle spectra).

The placement of P at position 6 of GlcN-(1-3) was established by a 2D ^1^H-^31^P HMBC experiment (**Appendix A**) that showed a connectivity of P (δP +1.2) to the assigned H-6,6′ resonances (δH 4.10–4.20) of GlcN-(1-3) (**Figure 2**; **Appendix A**). Evidence for the attachment of P to position 6 of GlcNAc-(1-2) was obtained from Smith degradation (NaIO_4_/NaBD_4_/H^+^) of wsPS-III that furnished ^2^H-Gro-(1-P-6)-α-GlcNAc-(1-2)-GroA. The ^1^H NMR spectrum of the transformation product showed the α-anomeric of GlcNAc-(1-2) at δH1 4.95 with the corresponding ring protons at δH2 3.99, δH3 3.86, δH4 3.61, δH5 3.96, and δH6,6′ 4.08–4.13 (**Figure 4**). The α proton (H-2) of GroA in this case resonated at δH 4.27 (not at δH 4.11 as seen in the native wsPS-III ^1^H NMR spectrum) and H-3 at δH 3.82-3.91 (**Figure 4**). ^1^H NMR studies at 315K and pH 5.5 did not result in a change in GroA H-2 chemical shift.

**Figure 5:**
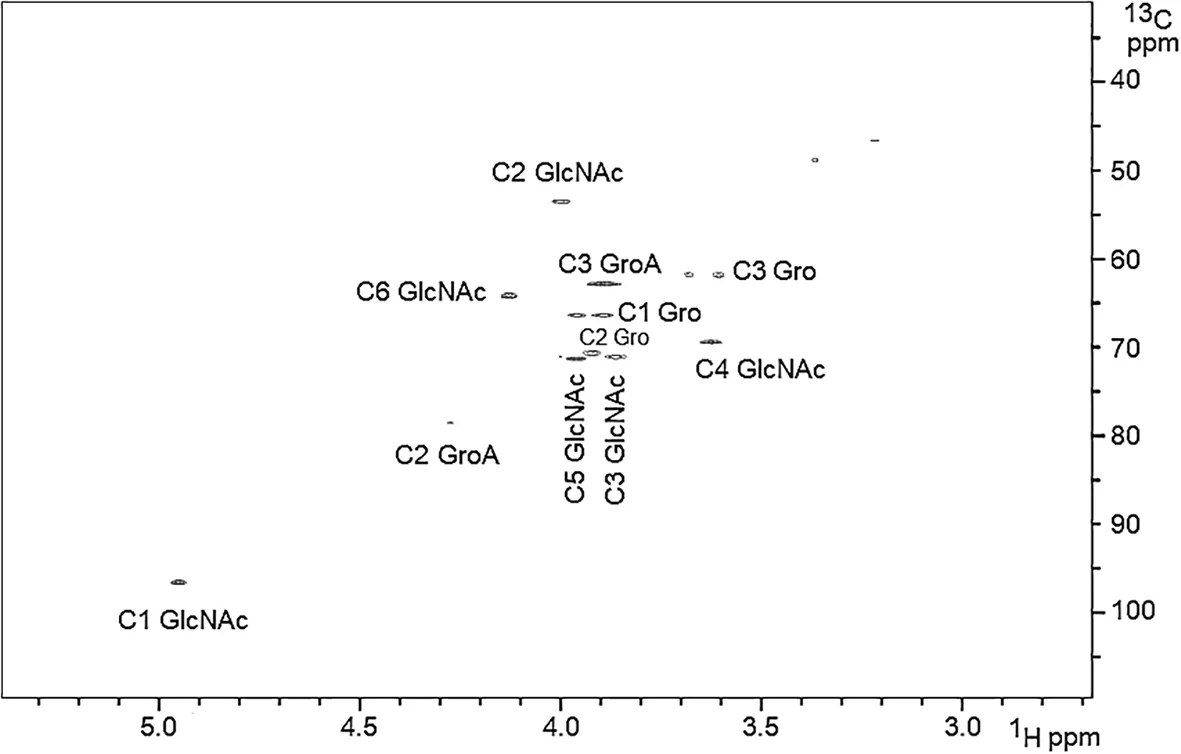
2D ^1^H-^13^C HSQC NMR spectrum of the Smith degradation product of wsPS-III, ^2^H-Gro-(1-P-6)-α-GlcNAc-(1-2)-GroA.

**Figure 5** displays the carbon resonances of the product from Smith degradation of wsPS-III, with those of GlcNAc-(1-2) at δC1 97.0, δC2 53.8, δC3 70.9, δC4 69.8, δC5 71.0, and δC6 64.1; those of GroA at δC2 77.6 and δC3 62.5; and those of the ^2^H-labeled glycerol, which originated from oxidation of P-6)-GlcN-(1-3), at δC1 66.0, δC2 70.5, and δC3 61.9. For ^2^H-Gro, two distinct H-1 resonances were associated with C-1 attached to the P moiety, at δH 3.89 and 3.95 (due to ^31^P coupling), and two distinct H-3 resonances associated with C3 at δH 3.60 and 3.69 (due to reduction of the aldehyde at C3 with NaBD_4_).

**Figure 6:**
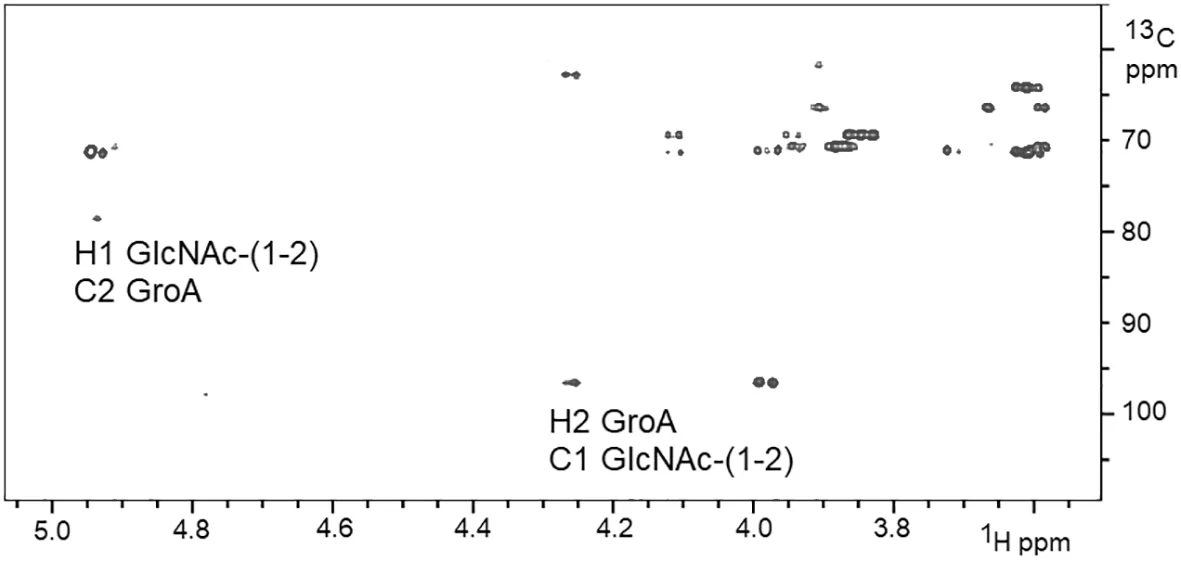
2D ^1^H-^13^C-HMBC NMR spectrum showing the GlcNAc-(1-2)-GroA linkage in the Smith degradation product ^2^H-Gro-(1-P-6)-α-GlcNAc-(1-2)-GroA.

**Figure 7:**
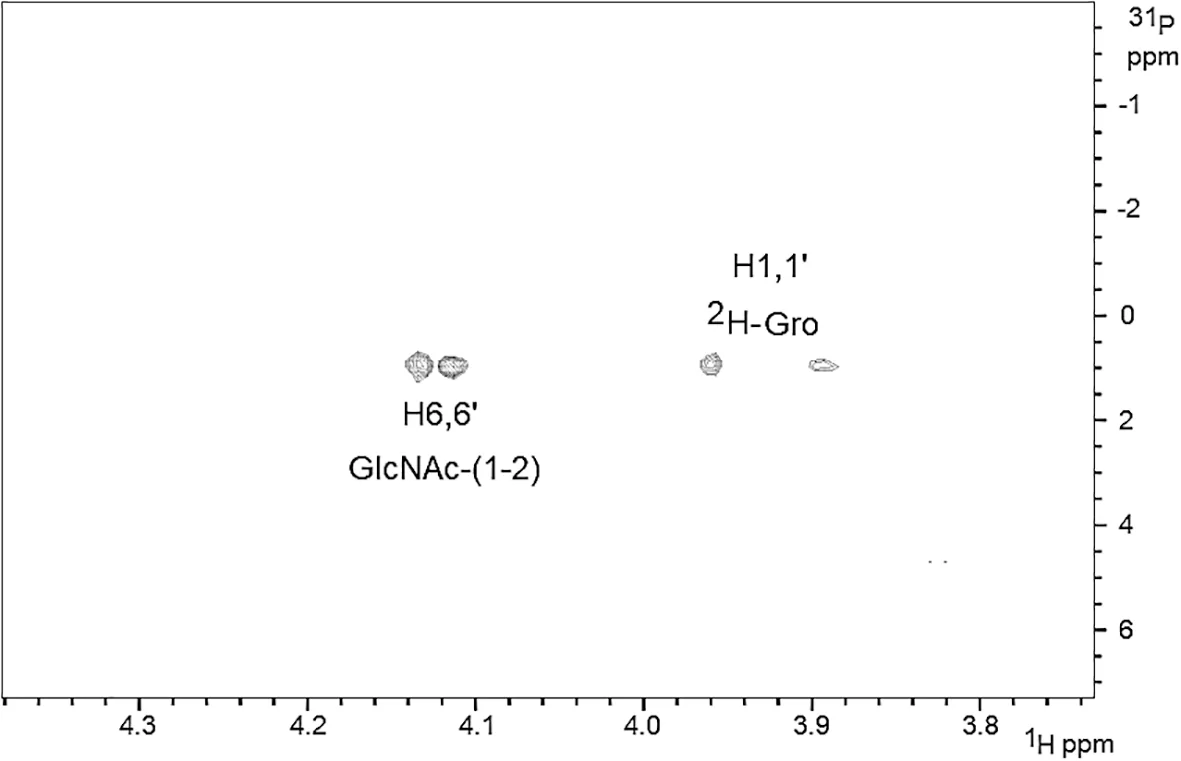
2D ^1^H-^31^P HMBC NMR spectrum of the Smith degradation product, ^2^H-Gro-(1-P-6)-α-GlcNAc-(1-2)-GroA, showing the placement of P between position 6 of GlcNAc-(1-2) and position 1 of ^2^H-Gro.

The linkage α-GlcNAc-(1-2)-GroA, in the transformation product ^2^H-Gro-(1-P-6)-α-GlcNAc-(1-2)-GroA, was confirmed by glycosidic linkage-associated inter H/C cross peaks in the 2D ^1^H-^13^C-HMBC NMR spectrum (**Figure 6**) at δH1 GlcNAc 4.95/ δC2 GroA 77.6 and δH2 GroA 4.27/ δC1 GlcNAc 97.0. The placement of the exocyclic P between C-1 of ^2^H-Gro and C6 of α-GlcNAc (1-2) was located through a 2D ^1^H-^31^P HMBC NMR experiment (**Figure 7**) that afforded resonances between the ^31^P signal and H-1,1′ of ^2^H-Gro and H6,6′ of GlcNAc-(1-2). The P unit in the transformation product ^2^H-Gro-(1-P-6)-α-GlcNAc-(1-2)-GroA resonated slightly upfield (δP +0.9) to that observed in the native wsPS-III (δP +1.2).

**Figure 8:**
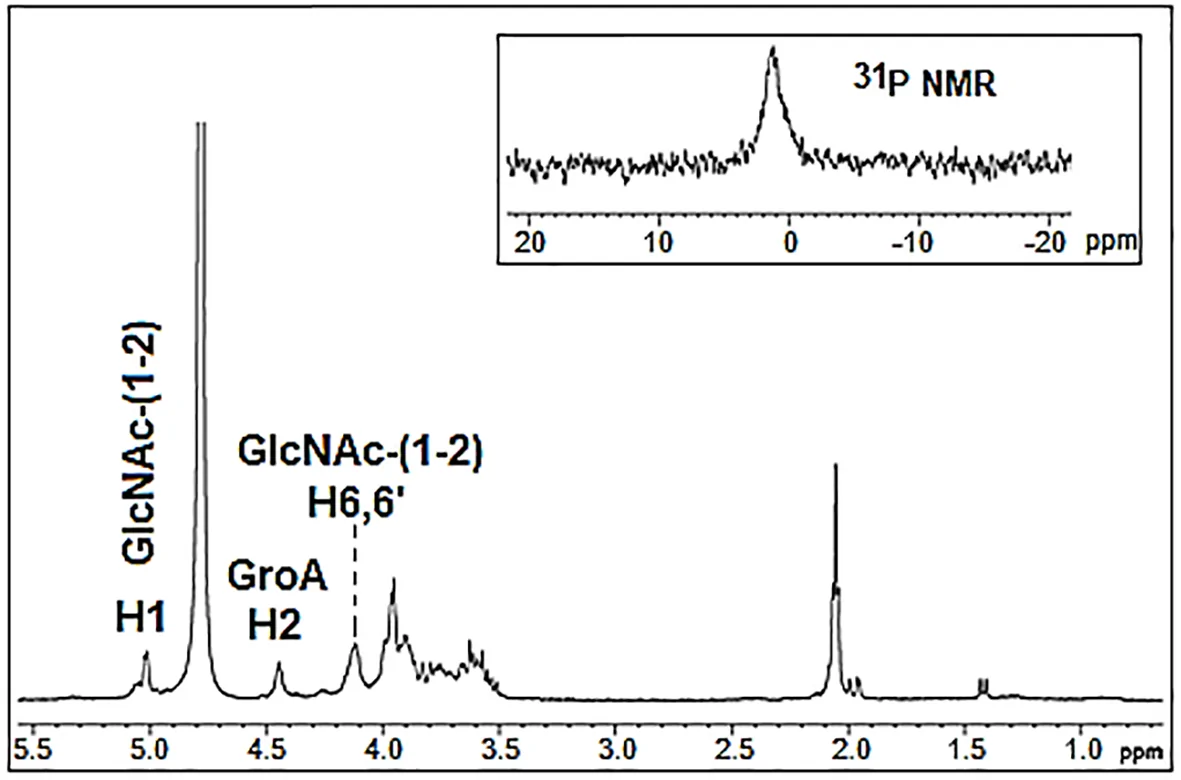
^1^H NMR and ^31^P NMR (inlet) spectra of Smith degradation product, ^2^H-Gro-(1-P-6)-α-GlcNAc-(1-2)-GroA, collected after colucharmn chromatography.

In the NMR spectra (**Figures 5**-**7**) of the transformation product described above, ^2^H-Gro-(1-P-6)-α-GlcNAc-(1-2)-GroA, which was purified by dialysis against water, the H-2 of GroA resonated at δH 4.27 and the H-1 of α-GlcNAc-(1-2) at δH 4.95. In a parallel experiment, in which the final product was collected after column chromatography, the H-2 of GroA resonated at δH 4.42 and the H-1 of α-GlcNAc-(1-2) at δH 5.00 (**Figure 8**).

**Figure 9:**
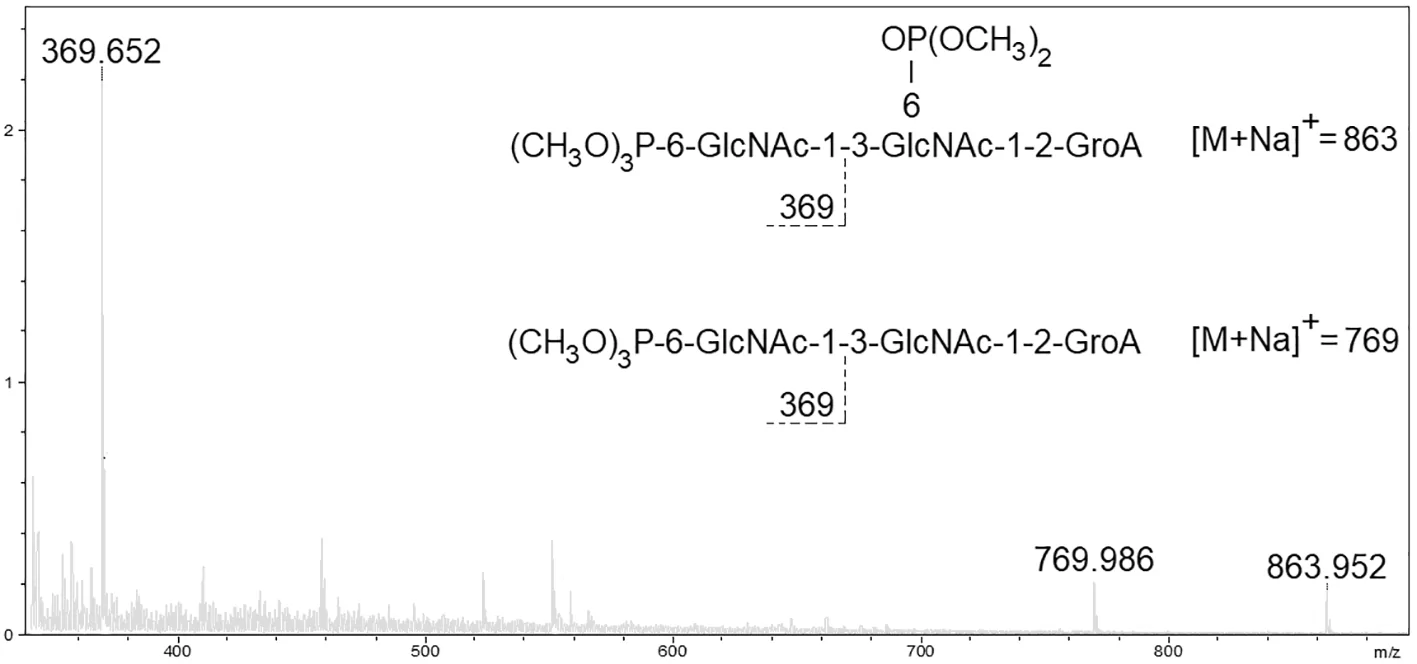
MALDI-ToF-MS of methylated wsPS-III.

A noteworthy set of data was obtained from the attempt to characterize the sugar linkage types through the methylation linkage analysis procedure (methylation/hydrolysis/acetylation). Due to the presence of the P diester, the expectations of characterizing any sugar linkages were low, and indeed, no permethylated alditol acetate derivatives were detected in GC–MS. However, MALDI-ToF-MS analysis of the methylated material (NaOH and CH_3_I in DMSO) yielded two ions separated by 94 amu (109 amu − 15 amu) corresponding to one di- O-methyl P monoester, OP(OCH_3_), at m/z 769 and 863 (**Figure 9**). m/z 863 was assigned to an oligosaccharide [M+Na]^+^ composed of one tri-O-methyl 6-GlcNAc (245 amu), one di-O-methyl 3,6-GlcNAc (230 amu), one di-O-methyl 2-GlcA (133 amu), one di-O-methyl P monoester OP(OCH_3_)_2_ (109 amu), and one tri-O-methyl phosphate monoester P(OCH_3_)_3_ (124 amu) (**Figure 9**). m/z 769 [M+Na]^+^ was assigned to a similar oligosaccharide, but lacking the di-O-methyl P monoester OP(OCH_3_)_2_, composed of one tri-O-methyl 6-GlcNAc (245 amu), one tri-O-methyl 3-GlcNAc (245 amu), one di-O-methyl 2-GlcA (133 amu), and one tri-O-methyl phosphate monoester P(OCH_3_)_3_ (124 amu). Evidence that the trimethyl P(OCH_3_)_3_ was attached at the monosubstituted 6-GlcNAc was revealed by the glycosyl oxonium ion at m/z 369 [P(OCH_3_)_3_-6-GlcNAc^+^] (124 amu + 245 amu) that originated from cleavage of the glycosidic bond between the two GlcNAc units. The detection of these two oligosaccharides revealed that the P diester bridging the two sugar units was hydrolyzed, as expected, under alkaline conditions. We propose that the pentavalent dimethyl P monoester, OP(OCH_3_)_2_, at position 6 of the 3,6-disubstituted GlcNAc, resulted from nucleophilic attack of the hydroxide ion from NaOH that leads to a P monoester dianion after elimination of the 6-monosubstituted GlcNAc. However, for the tetravalent trimethyl P monoester, P(CH_3_O)_3_, at position 6 of the 6-monosubstituted GlcNAc, it is hypothesized that the initial nucleophilic attack on phosphorus is carried out by the sugar alkoxide (O^–^ Na+) at position 4 of the 6-monosubstituted GlcNAc, forming a six-membered ring. Subsequent nucleophilic attack by the hydroxide ion from NaOH results in the elimination of the 3,6-disubstituted GlcNAc and the formation of a dianion-alcohol tetravalent P monoester that undergoes methylation to give P(CH_3_O)_3_. Note that the final location of the trimethyl P monoester P(CH_3_O)_3_, after opening of the six-membered ring, could be at the original position 6 or could have migrated to position 4 of the GlcNAc. No glycosyl oxonium ion at m/z 354 indicative of an OP(OCH_3_)_2_-6-GlcNAc^+^ was observed, and within the limits of detection, only ions associated with two GlcNAc units were seen in the MS spectrum.

### 3.2 Synthesis of α-D-GlcN-(1-3)-α D-GlcNAc-(1-2)-GroA and α-D-GlcNAc-(1-3)-α -D-GlcNAc-(1-2)-GroA

**Scheme 1:**
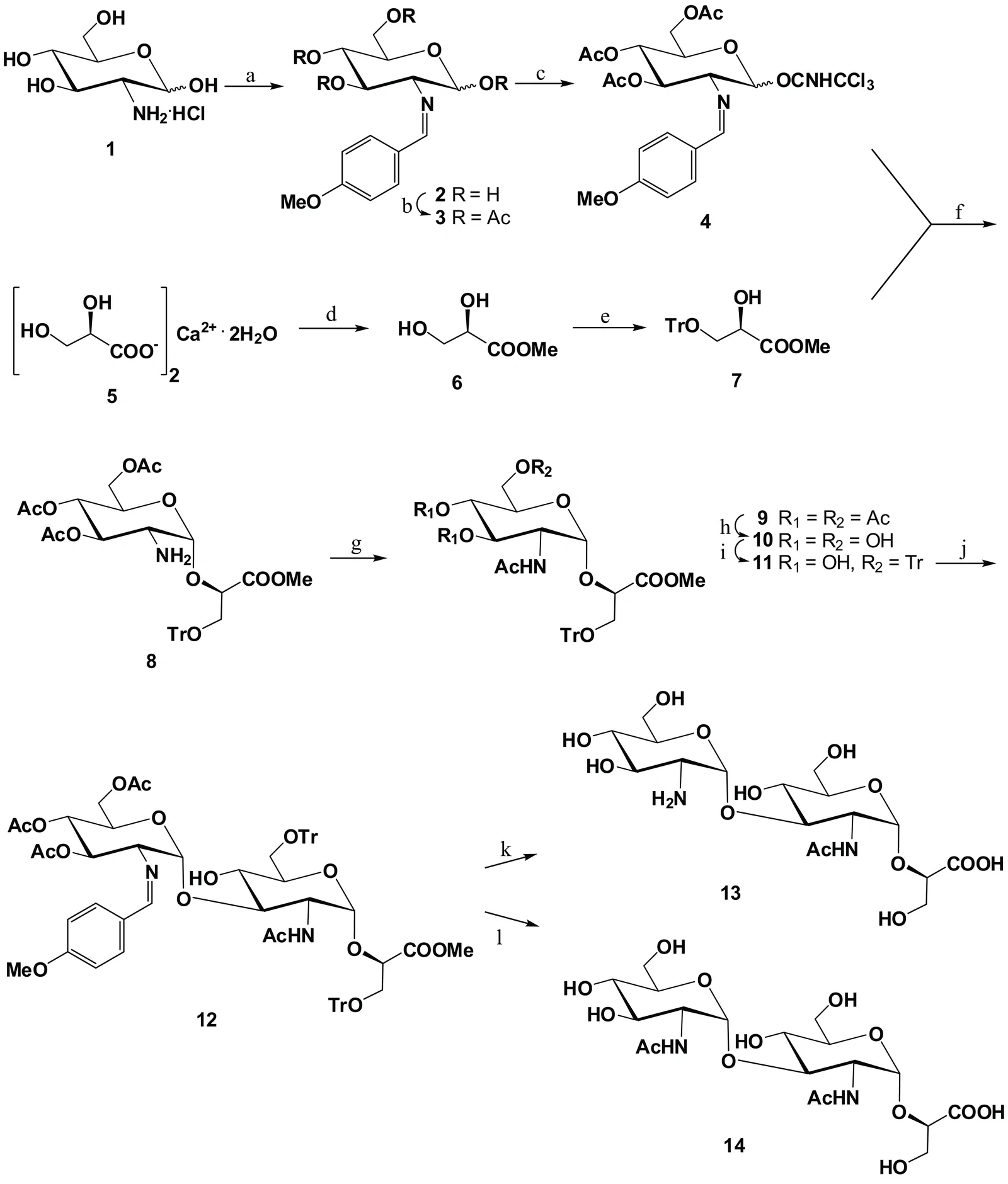
Reagent and conditions: (a) *p*-Anisaldehyde, NaOH (1 M), r.t.; (b) Ac_2_O, pyridine, r.t.; (c) i. NH_3_ in MeOH/toluene (1:3 v/v), r.t.; ii. Cl_3_CCN, K_2_CO_3_, CH_2_Cl_2,_ r.t., 62% starting from 1; (d) MeOH, 5% (v/v) AcCl, 0°C ~ r.t., 85%; (e) TrCl, pyridine, 55 °C, 78%; (f) AgOTf, MS, CH_2_Cl_2_, r.t., 67%; (g) Ac_2_O, pyridine, r.t., 98%; (h) K_2_CO_3_, MeOH, r.t., 77%; (i) TrCl, pyridine, 55 °C, 65%; (j) 4, AgOTf, MS, CH_2_Cl_2_, r.t., 55%; (k) i. HOAc 80%, 80 °C, ii. NaOMe, MeOH, r.t., 60% in two steps; (l) i. HOAc 80%, 80 °C, ii. Ac_2_O, pyridine, r.t., iii. NaOMe, MeOH, r.t., 57% in three steps.

Following the analysis of wsPS-III, the two related saccharides, α-D-GlcN-(1-3)-α-D-GlcNAc-(1-2)-GroA and α-D-GlcNAc-(1-3)-α-D-GlcNAc-(1-2)-GroA, were synthesized, mainly for comparison of NMR chemical shifts, specifically that of the α-proton (H-2) of GroA and H-1 of α-GlcNAc-(1-2), which were observed to differ in the glycans described above. During our structural analysis of wsPS-III, the syntheses of the phenol-soluble PS-III repeat containing two GlcNAc units, α-GlcNAc-(1-3)-α-GlcNAc-(1-2)-GroA, were reported (15, 37). These syntheses used the traditional azide group at C(2) position as a non-participating group for the formation of the 1,2-cis-2-amino glycosidic bond. Here, we describe an alternative method to synthesize the two saccharides blocks, α-GlcN-(1-3)-α-GlcNAc-(1-2)-GroA and α-GlcNAc-(1-3)-α-GlcNAc-(1-2)-GroA, in which the formation of the α-glucosamine linkage was achieved with a C(2) *para*-methoxy benzylidene-protected GlcN trichloroacetimidate as donor and silver triflate as promoter (**Scheme 1**).

Saccharides 13 and 14 (**Scheme 1**) were synthesized in a linear fashion, using the key building blocks GlcN donor 4 (36) and GroA acceptor 7, derived from GlcN and GroA, respectively. The 1,2-cis-glycosylation between donor 4 and acceptor 7 afforded product 8 in 67% yield, exclusively forming the α-glucosamine linkage. During this transformation, the para-methoxybenzylidene group was removed in situ. Whereas Nguyen reported the use of the cationic nickel(II) catalyst Ni(4-F-PhCN)_4_(OTf)_2_ for this reaction (36), silver triflate was found to provide comparable stereoselectivity under our conditions. Product 8 was then acetylated to give the N-acetamido-glucosyl glycerate 9 (GlcNAc-GroA) in 98% yield. Selective deacetylation of the O-3, O-4, and O-6 positions of 9 with potassium carbonate in methanol furnished compound 10 in 77% yield. Subsequent selective tritylation at O-6 afforded the GlcNAc-GroA acceptor 11 in 65% yield, leaving free hydroxyl groups at O-3 and O-4. Using conditions similar to those employed for the formation of 8, regioselective glycosylation of acceptor 11 with donor 4 occurred at O-3, delivering the fully protected saccharide repeating unit 12 (GlcN-GlcNAc-GroA) in 65% yield. Finally, 12 was treated with 80% aqueous acetic acid at 80 °C to remove acid-labile protecting groups, followed by sodium methoxide in methanol to give the zwitterionic target compound α-D-GlcN-(1-3)-α-D-GlcNAc-(1-2)-GroA (13) in 60% yield over two steps. Alternatively, acetylation prior to NaOMe/MeOH deprotection furnished α-D-GlcNAc-(1-3)-α-D-GlcNAc-(1-2)-GroA (14) in 57% yield over three steps.

**Figure 10:**
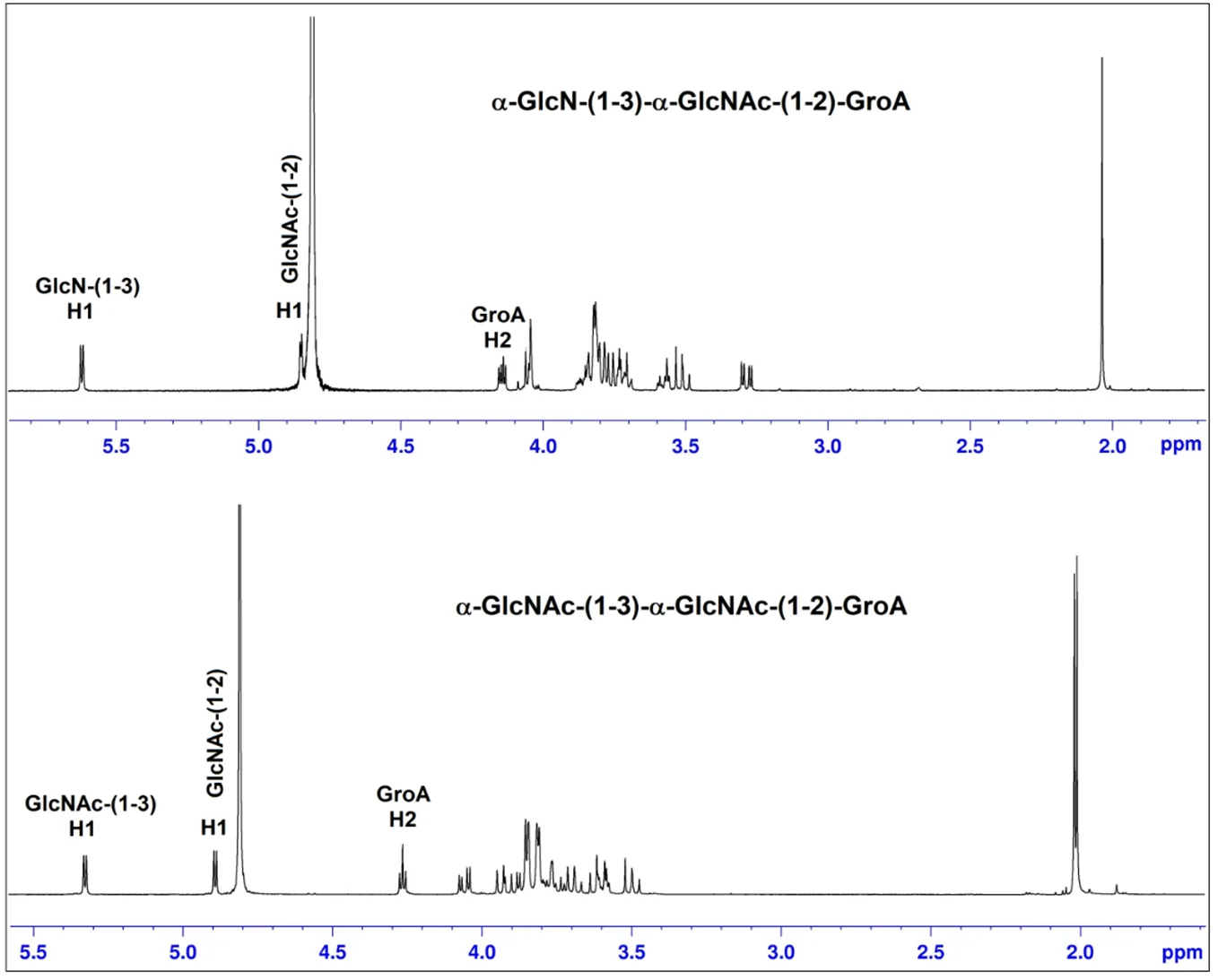
^1^H NMR spectra of synthetic products α-D-GlcN-(1-3)-α-D-GlcNAc-(1-2)-GroA (13) (top) and of α-D-GlcNAc-(1-3)-α-D-GlcNAc-(1-2)-GroA (14) (bottom).

The ^1^H NMR spectrum of α-D-GlcN-(1-3)-α-D-GlcNAc-(1-2)-GroA (13) showed that the H-2 of GroA resonated at δH 4.14 and H-1 of α-GlcNAc-(1-2) at δH 4.85 (**Figure 10**, **Table 1**). The H-2 of GroA and H-1 of α-GlcNAc-(1-2) of α-D-GlcNAc-(1-3)-α-D-GlcNAc-(1-2)-GroA (14) resonated at δH 4.27 and δH 4.90, respectively (**Figure 10**, **Table 1**). Previous work that described the syntheses of α-D-GlcNAc-(1-3)-α-D-GlcNAc-(1-2)-GroA reported the H-2 resonance of GroA at δH 4.26 and H-1 of α-GlcNAc-(1-2) at δH 4.90 (37) and at δH 4.46 for H-2 of GroA and H-1 of α-GlcNAc-(1-2) at δH 5.01 (15) (**Table 1**).

### 3.3 Immunodetection of wsPS-III IgG antibodies in sera of healthy horses and agglutination of *C.difficile* mediated by wsPS-III IgG antibodies

**Figure 11:**
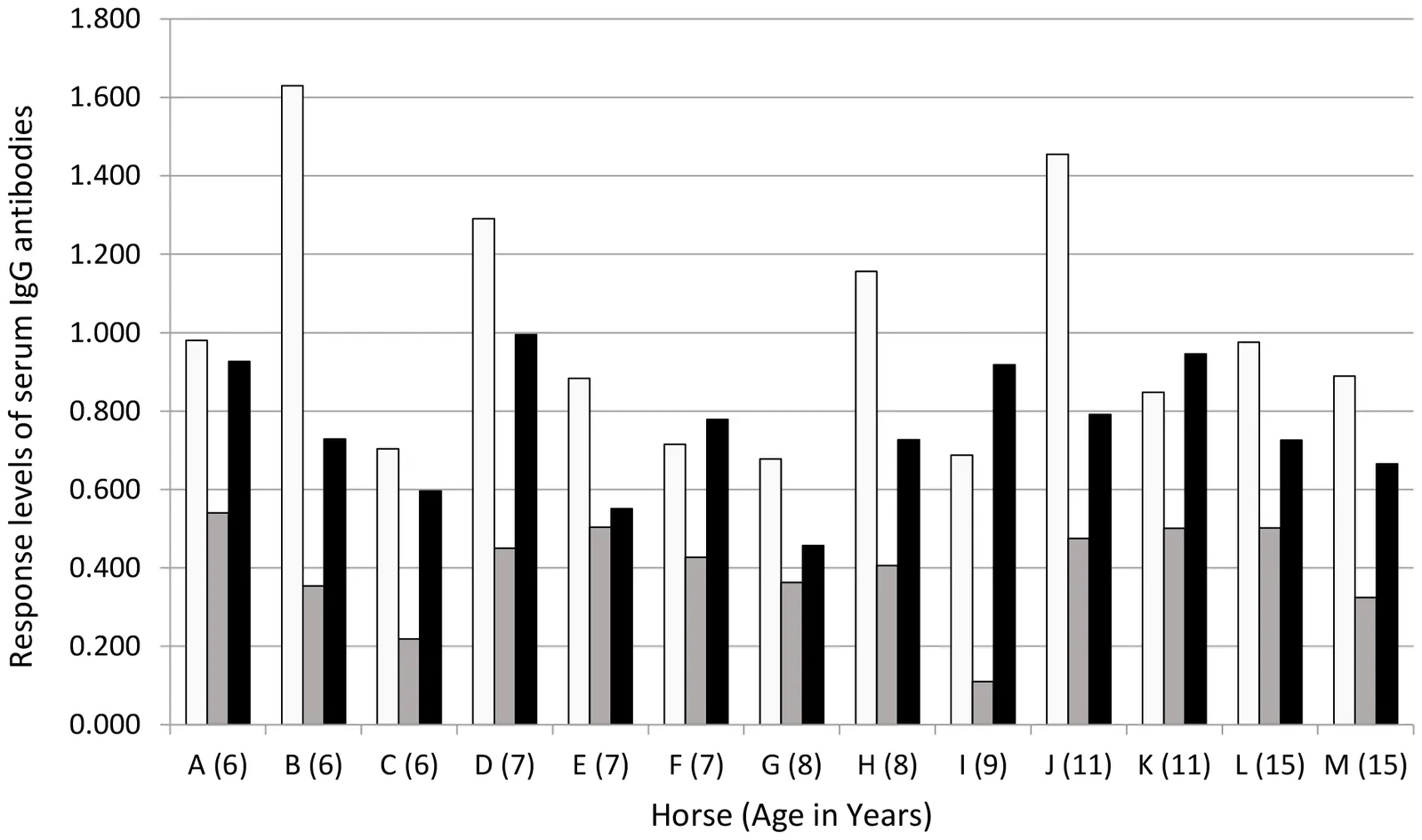
Responses of healthy horse serum IgG antibodies to *C. difficile* PS-I (white bars), PS-II (gray bars), and wsPS-III (black bars). Antibody concentrations are expressed as the ratio (S/P ratio) of the optical density for the serum sample to the optical density for a positive control serum diluted 1/50.

**Figure 12:**
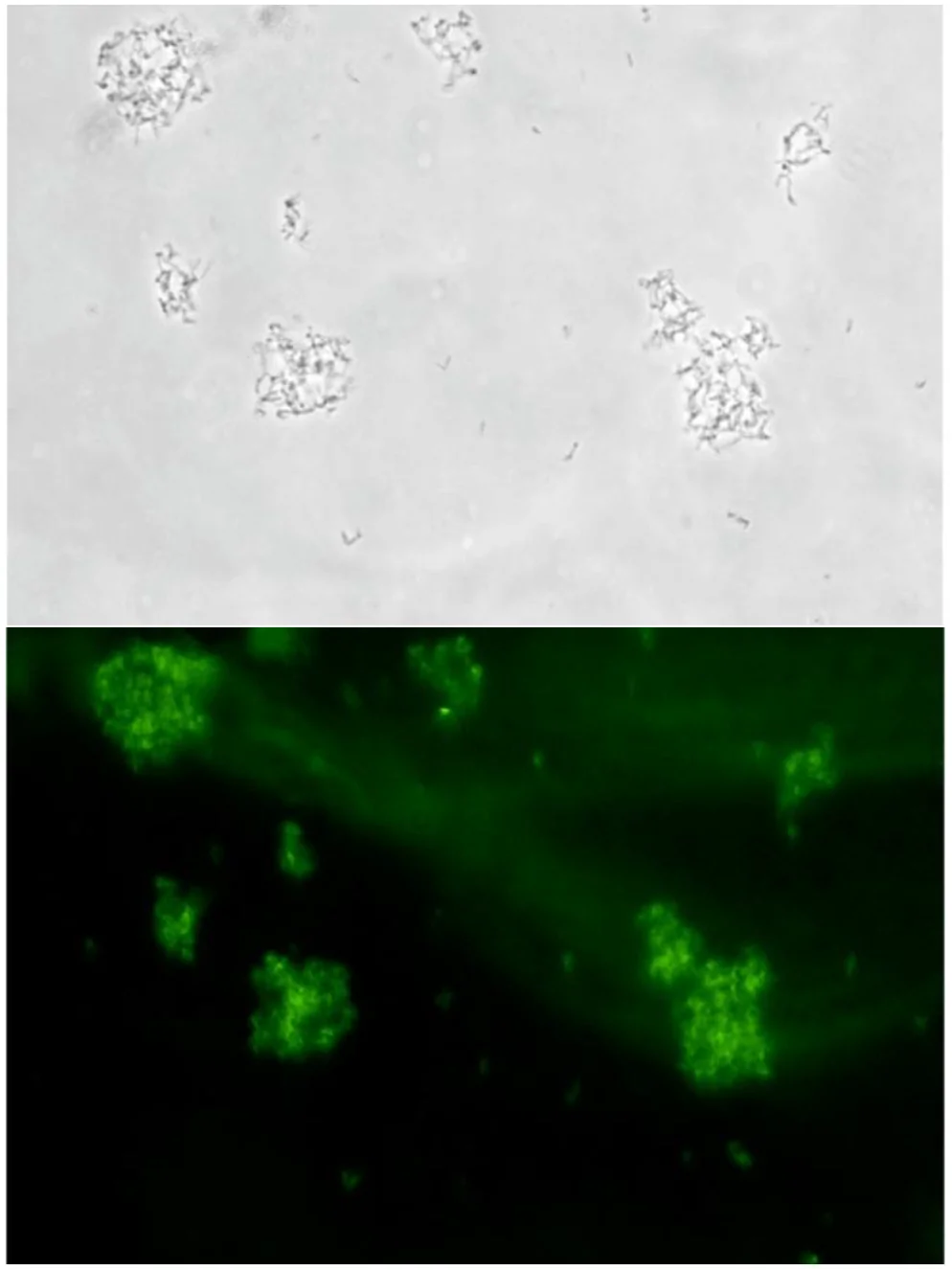
Immunofluorescence microscopy showing the reaction of wsPS-III IgG antibodies (raised in rabbits) with *C. difficile*.

Previously, we demonstrated that sera from healthy horses contained pre-existing circulating IgG antibodies that recognized *C. difficile* PS-I and PS-II (14, 16). Here, we show the reaction of the same healthy horse sera with *C. difficile* wsPS-III (**Figure 11**). All sera analyzed contained IgG antibodies that interacted with wsPS-III (black bars). Immunofluorescence microscopy revealed that immunization of rabbits with adjuvanted native wsPS-III produced IgG antibodies that reacted with *C. difficile* ribotype O27 strain (**Figure 12**).

## 4 Discussion

Collectively, the data showed that the polysaccharide component of *C. difficile* water-soluble LTA wsPS-III was mainly composed of repeating units [→PO_3_^−^-6)-α-D-GlcNH_3_^+^-(1-3)-α-D-GlcNAc-[1-2-GroA^−^]-(6→] contrary to the phenol-soluble LTA PS-III that was predominantly composed of [→PO_3_^−^-6)-α-D-GlcNAc-(1-3)-α-D-GlcNAc-[1-2-GroA^−^]-(6→] repeats (6, 7). The solubility of wsPS-III in water may be an effect of the high zwitterionic character of wsPS-III afforded by the presence of glucosamine.

The two saccharide repeating units α-D-GlcNH_3_^+^-(1-3)-α-D-GlcNAc-1-2-GroA^−^ (13) and α-D-GlcNAc-(1-3)-α-D-GlcNAc-1-2-GroA^−^ (14), containing α-2-amino/acetamido-glucosidic bonds, were efficiently synthesized through the combination of 2-*para*-methoxybenzylideneamino-2-deoxy-glucospyranosyl trichloroacetimidate as the donor and silver triflate as the promoter. Silver triflate required easy handling and proved to be an effective activator for the formation of 1,2-*cis*-2-amino/acetamido-glucosidic bonds.

An intriguing observation in this work was the chemical shift variability of H-2 of GroA and H-1 of α-D-GlcNAc-(1-2) in the NMR spectra of the saccharides analyzed (**Table 1**). In the glycans that contained GlcNH_3_^+^-(1-3), wsPS-III, and synthetic α-D-GlcN-(1-3)-α-D-GlcNAc-1-2-GroA 13, the H-2 of GroA resonated at δ 4.11 (**Figure 2**) and δ4.14 (**Figure 10**). In the case of glycans that contained GlcNAc-(1-3) in place of GlcNH_3_^+^-(1-3), the GroA H-2 resonated more downfield, that in PS-III at δ4.41 (6), and that of the synthetics D-GlcNAc-(1-3)-α-D-GlcNAc-1-2-GroA at δ 4.27 (**Figure 10**), δ4.26 (37), and δ 4.46 (15). To a lesser extent, the same pattern was observed for the H-1 of GlcNAc-(1-2) that is connected to position 2 of GroA, which resonated slightly more downfield in glycans without GlcNH_3_^+^-(1-3) (**Table 1**). There could be several reasons for the observed chemical shift variability of GroA H-2, such as concentration of samples, interaction of salts with the carboxyl of GroA, pH, or temperature. The different GroA H-2 chemical shifts seen between similar Smith degradation products (**Table 1**), at 4.27 (**Figure 4**) for the dialyzed product and at 4.42 (**Figure 8**) for the column purified one, suggested that the presence of salts may affect the chemical shift of GroA H-2. Likewise, the synthetic α-D-GlcNAc-(1-3)-α-D-GlcNAc-1-2-GroA previously described (15) that was also purified by column chromatography similarly showed the GroA H-2 resonance downfield at 4.46. We also propose here, based on ^1^H NMR data (**Figure 11**) of synthetics α-D-GlcNH_3_^+^-(1-3)-α-D-GlcNAc-1-2-GroA^−^ (13) and α-D-GlcNAc-(1-3)-α-D-GlcNAc-1-2-GroA^–^ (14), the possible formation of ammonium-glycerate salt-like complexes involving NH_3_^+^ of GlcN-(1-3) and COO^−^ of GroA in 13. These salt-like formations would induce a more upfield chemical shift for GroA H-2, at 4.14 in 13 and at 4.11 in wsPS-III. The presence of such ammonium-glycerate complexes could also in part explain the observed solubility of wsPS-III in water.

Naturally occurring circulating IgG antibodies in sera of healthy horses were found to react with wsPS-III (**Figure 11**). The presence of IgG antibodies in healthy horse sera specific to PS-I, PS-II, and wsPS-III points to the fact that exposure to *C. difficile* triggers an immune response against the surface PSs. S/P ratios were used in the current study to document the presence of antibodies in sera from naturally exposed horses. The horse serum standard used provides consistency for relative comparisons among sera for a fixed antigen but is not reliable for absolute comparisons between antigens.

Of particular significance was the reaction observed between wsPS-III IgG antibodies raised in rabbits by inoculation with sole adjuvanted wsPS-III without protein conjugation and *C. difficile* exposing PS-III (**Figure 12**). While direct T-cell activation profiles were not characterized in this study, the zwitterionic character of wsPS-III suggests that it may function as a T-cell-dependent antigen. We hypothesize that this molecular structure facilitates immune response elicitation via presentation by class II major histocompatibility complex (MHCII) molecules, a pathway observed in similar zwitterionic polysaccharides (38–44). However, given that the inoculation used complete Freund’s adjuvant that contains *M. tuberculosis* with extrinsic proteins and strong Toll-like receptor agonists, the possibility of a bystander T-cell help mechanism may also be a contributor to the observed IgG production.

The observed formation of clumps mediated by wsPS-III IgG antibodies indicates a potential neutralizing and clearing mechanism based on agglutination, likely leading to elimination through opsonization and phagocytosis (45, 46). To confirm this mechanism, future studies should employ T-cell proliferation assays and cytokine profiling to definitively establish the T-cell-dependent nature of the wsPS-III immune response.

This work revealed that an equine *C. difficile* strain expressed a water-soluble zwitterionic LTA composed mainly of repeating units with the GlcN-(1-3)-GlcNAc saccharide, [→P-6)-α-D-GlcN-(1-3)-α-D-GlcNAc-[1-2-GroA]-6→]. The first reported synthesis of the zwitterionic saccharide 13 repeat, α-D-GlcNH_3_^+^-(1-3)-α-D-GlcNAc-1-2-GroA^−^, was also described and shed light on possible reasons behind the observed variability in NMR chemical shifts of GroA H-2 and GlcNAc-(1-2) H-1. Insights into the immunogenic properties of wsPS-III were also gained through the detection of wsPS-III IgG antibodies in horse sera and agglutination of *C. difficile* by wsPS-III IgG antibodies raised by inoculation with sole wsPS-III.

## Data Availability

The original contributions presented in the study are included in the article/**Supplementary Material**. Further inquiries can be directed to the corresponding authors.
