# Supplementary material for: Clostridioides difficile water-soluble zwitterionic lipoteichoic acid wsPS-III: structure, synthesis, and immunogenicity
Source: Front Immunol. 2026 Aug 27;17:1901286. doi: 10.3389/fimmu.2026.1901286 (PMC13558421; doi:10.3389/fimmu.2026.1901286)
Supplement: Supplementary file 1 [file Table1.doc]

**Supplementary Spectral Data**

*Clostridioides difficile* water-soluble zwitterionic lipoteichoic-acid wsPS-III: Structure, synthesis and immunogenicity

Zuchao Ma^a,^*, Brittany Pequegnat^a^, Douglas Hodgins^b^, Martin Sagermann^c^, Jeyabarathy Ganeshapillai^a^, Sharif Arar^a^, Jamie Dawson^a^, Nolan W. Frame^a^, Samantha Wheadon^a^, Herbert Chow^c^, Luis Arroyo^d^ and Mario A. Monteiro^a,^*

^a^*Department of Chemistry, University of Guelph, Guelph, ON, Canada*

^b^*Department of Pathobiology, University of Guelph, Guelph, ON, Canada*

^c^*Stellar Biotechnologies Inc., CA, USA*

^d^*Department of Clinical Studies, University of Guelph, Guelph, ON, Canada*

**NMR spectra of new synthetic compounds:**

Compound	Page

**4**…………………………..2

**7**…………………………..3

**8**…………………………..4

**9**………………………..…5

**10**………………………....6

**11**…………………………7

**12**………………………....8

**13**…………………………9

**14**………………………....10

**Figures of:** Page

1D ^1^H-^1^H TOCSY NMR spectra showing the assignments of H-1 to H-6 of

GlcN-(1-3); irradiation of H-1 (A) and H-2 (B)…………………………………..…….11

2D ^1^H-^31^P HMBC NMR spectrum of wsPS-III showing a connection between

P (δ_P_ +1.2) and H6,6’ (δ_H_ 4.10-4.20) of GlcN-(1-3) for a P-6)-GlcN-(1-3) linkage...….12

**4**: ^1^H NMR (CDCl3, 400 MHz)

**4**: ^13^C NMR (CDCl3, 100 MHz)

**7**: ^1^H NMR (CDCl3, 400 MHz)

**7**: ^13^C NMR (CDCl3, 100 MHz)

**8**: ^1^H NMR (CDCl3, 400 MHz)

**8**: ^13^C NMR (CDCl3, 100 MHz)

**9**: ^1^H NMR (CDCl3, 400 MHz)

**9**: ^13^C NMR (CDCl3, 100 MHz)

**10**: ^1^H NMR (CDCl3, 400 MHz)

**10**: ^13^C NMR (CDCl3, 100 MHz)

**11**: ^1^H NMR (CDCl3, 400 MHz)

**11**: ^13^C NMR (CDCl3, 100 MHz)

**12**: ^1^H NMR (CDCl3, 400 MHz)

**12**: ^13^C NMR (CDCl3, 100 MHz)

**13**: ^1^H NMR (D_2_O, 400 MHz)

**13**: ^13^C NMR (D_2_O, 100 MHz)

**14**: ^1^H NMR (D_2_O, 400 MHz)

**14**: ^13^C NMR (D_2_O, 100 MHz)

1D ^1^H-^1^H TOCSY NMR spectra showing the assignments of H-1 to H-6 of GlcN-(1-3); irradiation of H-1 (A) and H-2 (B).

2D ^1^H-^31^P HMBC NMR spectrum of wsPS-III showing a connection between P (δ_P_ +1.2) and H6,6’ (δ_H_ 4.10-4.20) of GlcN-(1-3) for a P-6)-GlcN-(1-3) linkage.
